# DORQ-seq: high-throughput quantification of femtomol tRNA pools by combination of cDNA hybridization and Deep sequencing

**DOI:** 10.1093/nar/gkae765

**Published:** 2024-09-11

**Authors:** Marco Kristen, Marc Lander, Lea-Marie Kilz, Lukas Gleue, Marko Jörg, Damien Bregeon, Djemel Hamdane, Virginie Marchand, Yuri Motorin, Kristina Friedland, Mark Helm

**Affiliations:** Institute of Pharmaceutical and Biomedical Sciences, Johannes Gutenberg University Mainz, Staudingerweg 5, 55128 Mainz, Germany; Institute of Pharmaceutical and Biomedical Sciences, Johannes Gutenberg University Mainz, Staudingerweg 5, 55128 Mainz, Germany; Institute of Pharmaceutical and Biomedical Sciences, Johannes Gutenberg University Mainz, Staudingerweg 5, 55128 Mainz, Germany; Institute of Pharmaceutical and Biomedical Sciences, Johannes Gutenberg University Mainz, Staudingerweg 5, 55128 Mainz, Germany; Institute of Pharmaceutical and Biomedical Sciences, Johannes Gutenberg University Mainz, Staudingerweg 5, 55128 Mainz, Germany; IBPS, Biology of Aging and Adaptation, Sorbonne Université, Paris 75252, France; Laboratoire de Chimie des Processus Biologiques, CNRS-UMR 8229, Collège De France, Université Pierre et Marie Curie, 11 place Marcelin Berthelot, 75231 Paris, Cedex 05, France; Université de Lorraine, IMoPA UMR7365 CNRS-UL, BioPole, 54000 Nancy, France; Université de Lorraine, IMoPA UMR7365 CNRS-UL, BioPole, 54000 Nancy, France; Université de Lorraine, Epitranscriptomics and RNA Sequencing (EpiRNA-Seq) Core Facility, UAR2008 IBSLor (CNRS-UL)/US40 (INSERM), 54000 Nancy, France; Institute of Pharmaceutical and Biomedical Sciences, Johannes Gutenberg University Mainz, Staudingerweg 5, 55128 Mainz, Germany; Institute of Pharmaceutical and Biomedical Sciences, Johannes Gutenberg University Mainz, Staudingerweg 5, 55128 Mainz, Germany

## Abstract

Due to its high modification content tRNAs are notoriously hard to quantify by reverse transcription and RNAseq. Bypassing numerous biases resulting from concatenation of enzymatic treatments, we here report a hybrid approach that harnesses the advantages of hybridization-based and deep sequencing–based approaches. The method renders obsolete any RNAseq related workarounds and correction factors that affect accuracy, sensitivity, and turnaround time. Rather than by reverse transcription, quantitative information on the isoacceptor composition of a tRNA pool is transferred to a cDNA mixture in a single step procedure, thereby omitting all enzymatic conversations except for the subsequent barcoding PCR. As a result, a detailed tRNA composition matrix can be obtained from femtomolar amounts of total tRNA. The method is fast, low in cost, and its bioinformatic data workup surprisingly simple. These properties make the approach amenable to high-throughput investigations including clinical samples, as we have demonstrated by application to a collection of variegated biological questions, each answered with novel findings. These include tRNA pool quantification of polysome-bound tRNA, of tRNA modification knockout strains under stress conditions, and of Alzheimer patients’ brain tissues.

## Graphical Abstract

**Graphical Abstract:**
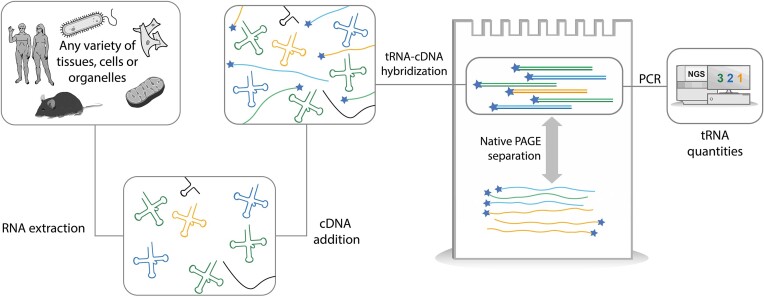

## Introduction

Transfer RNAs (tRNAs) are universally expressed small non-coding RNA molecules, bearing a distinct secondary structure and extensive levels of RNA modifications. tRNAs are crucial for protein biosynthesis in translating nucleic acid encoded information from messenger RNA (mRNA) to functional proteins. Alterations in the process of mRNA translation have been associated with various diseases in humans, comprising neurological disorders and cancer (1,2). Approaches to quantify these mRNAs in order to obtain gene expression profiles have recently proven insufficient on their own, missing impact of numerous post-transcriptional factors. At this point, ribosomes, the key player carrying out protein synthesis itself, are highly dependent on availability and abundance of tRNAs, thereby directly influencing speed and efficiency of protein synthesis (3,4). Considering the diverse pool of tRNAs, differing in cognate amino acid and dynamic expression, an accurate, and experimentally simple method for tRNA pool quantification is still highly desirable in biochemical and biomedical sciences.

Despite recent advances, the research field currently lacks an easily accessible and precise high-throughput method for fast and inexpensive characterization of tRNA pools from smallest tRNA quantities. Current methods based on gel electrophoresis offer acceptable accuracy in exchange for the significant effort and time consumption to prepare them, even for single tRNA subtypes. Methods utilizing the distinct sequence of every tRNA subtype to specifically target single tRNAs with an individually tailored nucleic acid counterpart (oligonucleotide), so-called tRNA microarrays, require tremendous hands-on effort and are limited by their fluorescence-based detection (5,6). Due to such restrictions, only few different samples can be analyzed for their full tRNA pool per microarray, thus limiting throughput-capability and therefore applicability for experiments with numerous conditions and replicates. Over the last decade, methodical developments capitalized on high throughput methods, chiefly on Illumina-based next generation sequencing (NGS), allowing to analyze even very rare tRNA subtypes (7,8). Because this method is unable to directly analyze the tRNAs, pertinent RNAseq protocols in general require numerous enzymatic steps during sample preparation. Generally, RNA must be converted to DNA (reverse transcription), a step greatly impeded by structural peculiarities of tRNAs, such as the extensive secondary structure and a vast repertoire of over 160 modified ribonucleotides, each of them idiosyncratically interfering with complete and quantitative reverse transcription. Next, a so-called adapter ligation, used in most methods and based on enzymatic or chemical treatment, varies in efficiency, introducing selection bias and increasing the required amount of tRNA for the method. The same applies to further steps, including the final polymerase chain reaction (PCR) which is required to introduce recognition sequences essential to NGS. The latter may display varying amplification efficiencies and may cause further quantitative bias.

Another important factor is the required input amount of tRNA which ranges around 1–2 μg for microarrays and gel-based approaches (2,9). This amount can be limiting in terms of preparative effort to acquire enough tRNA or biological availability, e.g. when analyzing minor tissue compartments, not yielding sufficient tRNA quantities due to their small size. Current NGS methods lowered the barrier in terms of input requirements, normally ranging between 50 and 500 ng per sample (7,8), an amount achievable in many preparations, however still at the limit of what is feasible when collecting rare or small cells/tissues and considering technical replicates. One more detrimental step in RNAseq based tRNA quantification is the bioinformatic workup of large datasets, which consistently requires sustained user training and is liable to consume significant time and effort.

Clearly, there is a sustained demand for tRNA quantification, as evidenced by the numerous approaches recently developed. While earlier work used yeast tRNAs as a gold standard, more recent methods focus on mammalian tRNA populations, as we will discuss later. Hence, owing in part to these limitations in publicly available datasets, a comprehensive comparison is difficult.

In view of the various obstacles encountered in tRNA quantification, we developed a novel method combining a cDNA hybridization technique with next generation sequencing, thus overcoming RT-derived hindrances while achieving high-throughput capability. We targeted individual tRNA isoacceptors by designing complementary DNA oligonucleotides, the latter already containing adapter sequences required for final library preparation. A tRNA-cDNA hybridization step transfers quantitative information to the cDNA template, thus omitting reverse transcription and any enzymatic step except for a low-cycle index PCR which takes place on the cDNA template. Following NGS, the limited pool of unmodified cDNA reference sequences allows for a greatly simplified bioinformatic analysis which is, in comparison to any other RNAseq approach, almost trivial and therefore extremely fast. Furthermore, preparational effort for 96 samples comprises only around 5 days with 15–20 h of hands-on time and a total cost below 50$ per sample, considering consumables and NGS, which can be outsourced. Finally, our method allows for tRNA pool characterization with down to 5 ng of tRNA, lowering the RNA input requirements by one order of magnitude compared to current RNAseq methods.

## Material and methods

### Cell line samples

HEK293- (DSMZ, Braunschweig, Germany, No°ACC 305), HeLa- (DSMZ, No°ACC 57) and T98g- (ATCC, Manassas, VA, USA, No°CRL-1690) cells were cultivated in Dulbecco's modified Eagle's medium (Thermo Fisher Scientific, Waltham, MA, USA) supplemented with 10 vol-% fetal bovine serum (Thermo Fisher Scientific) and 0.1% Primocin (InvivoGen, San Diego, CA, USA), GlutaMAX™ (Thermo Fisher Scientific), 4.5 g/l glucose (Thermo Fisher Scientific) and sodium pyruvate (Thermo Fisher Scientific) at 37°C and 5 vol% CO_2_. For separation or harvesting, cells were first washed with Dulbecco's phosphate-buffered saline (Thermo Fisher Scientific) and then incubated with trypsin-EDTA (Thermo Fisher Scientific) for 5 min under growing conditions. After detaching, cells were centrifuged for 5 min at 400 g, washed with phosphate buffer and once again centrifuged. Finally, pellets were dissolved in TRI Reagent (Sigma-Aldrich, St. Louis, MO, USA) and stored at −20°C. Similarly, *S. cerevisiae* was cultivated in YPD-medium (Carl-Roth, Karlsruhe, Germany) up to an OD of 1.0, centrifuged at 10 000 g for 20 min, supernatant decanted, the cell pellet dissolved in TRI Reagent and stored at −20°C.

### Murine tissue samples

Female C57BL/6J mice were used at an age of 16 weeks (for housing conditions and genotyping see Brandscheid *et al.* (10)). All experimental procedures were carried out in accordance with the European Communities Council Directive regarding care and use of animals for experimental procedures and was approved by local authorities (LUA-Rhineland-Palatinate).

### Human tissue samples

Human brain samples were provided by the rapid autopsy program of the Netherlands Brain Bank (NBB, Amsterdam, Netherlands) for high quality specimen from clinically well-documented and neuropathologically confirmed cases. Research on human specimens was performed according to the ethical declaration of the NBB. All cases of Alzheimer's disease were neuropathologically confirmed using the Consortium to Establish a Registry for Alzheimer's Disease (CERAD) criteria. The control specimen had no history or symptoms of neurologic or psychiatric disorders and showed no clinical symptoms of dementia. Tissue from the Gyrus frontalis superior 3 + 4 was used for all 12 control and 13 AD specimens. Age varied between 72 to 98 years for AD patients (average 84) and 71 to 102 for control patients (average 85), with an average Braak stage of 4.7 (AD) or 2.5 (Ctr).

### RNA isolation from cells or tissue

Total RNA was isolated from either cell pellets or dissected tissue by extraction with TRI Reagent (Sigma-Aldrich). The cell pellet (∼2 000 000 cells) or tissue (∼50 mg) was suspended in 2 ml TRI Reagent and thoroughly mixed, in case of tissue also crushed with a pestle. Following addition of 400 μl chloroform (Carl Roth) and thorough mixing, samples were centrifuged at 18 000 g for 15 minutes, the aqueous phase transferred to a new tube and 1 ml 2-propanol (Carl Roth), as well as 1 μl Glycogen (Thermo Fisher Scientific), were added. Following another round of centrifugation at 18 000 g for 15 min, supernatant was removed and 1 ml ethanol (75%) (Thermo Fisher Scientific) was added for a final centrifugation step. Finally, supernatant was carefully removed, the RNA pellet briefly airdried and resuspended in RNase free water. The required amount of TRI Reagent varied depending on the number of cells or tissue size used, with respective changes to the other Reagents that were added.

### Polysome profiling and tRNA isolation

The *E. coli* wildtype strain Keio parent (BW25113) and the knockout strains *ΔdusA* and *ΔdusB* were purchased from the *E. coli* Keio knockout collection (GE Healthcare (DharmaconTM, England). *E. coli* cultures were grown in 100 ml LB medium (Carl Roth) at 37°C and 190 rpm until an optical density of 0.4 at 600 nm. Paraquat dichloride hydrate (Sigma-Aldrich) was added to final concentrations of 0, 0.1 and 0.3 mM and bacterial growth was continued until an OD_600_ of 0.7. Chloramphenicol (100 μg/ml, Carl Roth) was added to the culture which was incubated for 3 min and then harvested by centrifugation (10 min, 10 000 g, 4°C). The pelleted cells were resuspended in buffer (100 mM NH_4_Cl (Merck, Darmstadt, Germany), 10 mM MgCl_2_ (Carl Roth), 20 mM Tris, pH 7.5 (Carl Roth)), lysozyme (Carl Roth) was added and freeze-thaw cycles in liquid nitrogen were performed. Lysis was completed by adding 10% deoxycholate (Sigma-Aldrich) and cell wall debris was removed by centrifugation (12 000 g, 10 min, 4°C). Cell lysate was loaded on top of sucrose gradients from 5 to 40% using Biocomp (BioComp, Fredericton, Canada) gradient station model 108 (settings: time 1.23 min, angle 81.5 °, speed 21 rpm). Gradients were ultracentrifuged (150 000 g, 4°C, 2.5 h, Beckman Optima MAX-XP Ultracentrifuge, SW40 Ti rotor from Beckman Coulter, CA, USA) and fractionated collecting free RNA fraction (F0) and polysomal fraction (F3). Total RNA was extracted using TRI Reagent (Sigma-Aldrich) and separated subsequently on a 10% denaturing PAGE gel stained with GelRed (Biotium/BIOTREND, Köln, Germany). tRNA bands were excised from the gel after visualization of RNA bands using Typhoon 9400 (excitation wavelength of 532 nm, Amersham Bioscience/GE Healthcare, Chicago, IL, USA) and mashed with a scalpel. tRNA was extracted from the gel adding 300 μl 0.5 M ammonium acetate (Merck) before overnight incubation at 25°C and 750 rpm. After filtration through NanoSep 0.45 μM spin columns (VWR, Darmstadt, Germany), RNA was precipitated using three volumes of 100% ethanol (Carl Roth).

### cDOQ design

cDNA oligonucleotides for quantification (cDOQs) were designed from tRNA references retrieved from tRNAdb (11), MODOMICS (12), and gtRNAdb (13). The last 40 nucleotides towards the tRNA 3′ end were compiled to non-redundant references utilizing a R-based construction pipeline by Pichot *et al.* (14), collapsing sequences with a levenshtein distance below six nucleotides into one species (Supplementary Figure S1B). Next, the reverse-complement of these retrieved, non-redundant references was merged with the respective P5 (AGACGTGTGCTCTTCCGATCT) and P3 (GATCGTCGGACTGTAGAACTCTGAAC) sequence on the 5′ and 3′ end. The full-length constructs of 87 nucleotides were finalized with either a 6-FAM (cytosolic tRNAs) or Cyanine-5 (mitochondrial tRNAs) attached to the 5′ end. All cDOQs were ordered and synthesized at biomers.net GmbH (Ulm, Germany) or Integrated DNA Technologies (Coralville, IA, USA).

### PCR and sequencing primer design

For indexing PCR, twelve i7 and eight i5 primers were designed to allow multiplexing up to 96 samples. To reach full compatibility with Illumina platforms, a custom primer for the i5 index read on Illumina NextSeq platforms was designed and utilized on the sequencing platform. All Primers were ordered and synthesized at biomers.net GmbH.

### cDOQ-tRNA hybridization and hybrid purification

Starting from 50–200 ng of total RNA or 5–50 ng of total tRNA, 5x hybridization buffer (150 mM HEPES pH 7.5 (Carl Roth), 500 mM potassium acetate (Carl Roth)) was added. Following addition of a 5-fold excess of an equimolar mixture of cDOQs for the selected targets (e.g. a single tRNA or 43 cytosolic + 22 mitochondrial tRNAs in human), the solution was denatured at 94°C for 2 min and afterwards gradually cooled down to 25°C within 20 min. The sample was then analyzed on a 10% native polyacrylamide-gel in 1x TBE buffer, running at constant power of 60 mA for 45 min. Depending on the experimental context, optional GelRed-staining (Biotium, Fremont, CA, USA) was carried out for 15 min to visualize nucleic acids. cDOQ-attached fluorescent dyes used for stainless visualization comprised 6-FAM or Cy5. Bands of interest (e.g. cDOQ:tRNA hybrid) were excised, crushed and eluted overnight in 300 μl 0.5 M ammonium acetate (Merck) at 750 rpm and 15°C. Afterwards, gel particles were removed with Nanosep 0.45 μM spin columns (VWR), centrifuging at 2000 g for 1 min. Finally, 1 μl glycogen was added to the aqueous flow-through, together with 2.5 volumes of ethanol (99,5%, Carl Roth), storing the mixture at −80°C for 1 h or −20°C overnight. Afterwards, samples were centrifuged at 18 000 g for 1 h, supernatant removed, the pellet washed with 500 μl ethanol (70%) and again centrifugated at 18 000 g for 30 min. Supernatant was carefully removed, the pellet left to air-dry for 10 min at room temperature and then resuspended in 10 μl of RNAse-free water.

### Indexing PCR and next generation sequencing

Nucleic acids from excised cDOQ:tRNA hybrids were subjected to index PCR, using half (5 μl) of the previously purified product. For samples with low tRNA input (5 ng or less), complete use of retrieved product was necessary. From a master mix, Taq-Buffer (NEB, Frankfurt am Main, Germany), dNTPs (NEB) and MgCl_2_ (Carl Roth) were added to final concentrations of 1× (Buffer), 0.5 mM (dNTP) and 3 mM (MgCl_2_). Individual i5 and i7 primer combinations were added to a final concentration of 0.25 μM. Reaction was carried out with Taq-Polymerase (NEB), initially denaturing at 94°C for 30 s, followed by normally 3–6 cycles of denaturation (94°C, 15 s), annealing (62°C, 30 s) and extension (72°C, 15 s), as well as a final extension step at 70°C for 5 min. Next, the reaction mixture was purified on a denaturing 10% PAGE in 1× TBE buffer, running at constant power of 60 mA for 1 hour, excising the product band around 169 nt length. Following overnight elution, ethanol precipitation and resuspension as described previously, samples were sequenced on an Illumina NextSeq 2000 or MiSeq platform. For sequencing on the NextSeq 2000 platform, a custom i5 index read primer (Supplementary Table S1) was used. The achieved read output proved to be dependent on the number of different samples pooled on a flow cell, as well as the amount of sequencing-ready library loaded for each sample. Thus, for samples with low tRNA input (5 ng or less), yielding less DNA in the low-cycle index PCR, adjustments in library amount used and the overall sample number loaded on the flow-cell had to be considered.

### RNAseq

Library preparation of tRNA fractions was based on the NEBnext Small RNA Library Prep Set for Illumina (NEB) and is briefly described below. First, tRNA was dephosphorylated with Antarctic Phosphatase (NEB) and afterwards phosphorylated with Polynucleotide Kinase (NEB). Following purification via RNeasy MinElute Cleanup kit (Qiagen, Hilden, Germany), a preadenylated 3′ adaptor was ligated and the reverse transcription primer hybridized to minimize adaptor-dimer formation. Next, the 5′ adaptor was ligated and reverse transcription was performed using SuperScript IV (Thermo Fisher Scientific) for its high sequence fidelity and ability to process sites comprising RNA modifications. Finally, polymerase chain reaction was performed using custom i5 and i7 indexes (Supplementary Table S1) and the PCR product purified via gel excision on a denaturing polyacrylamide gel. Full length product was then subjected to sequencing on an Illumina NextSeq 2000 platform using a custom i5 index read primer (GATCGTCGGACTGTAGAACTCTGAAC).

### NGS data analysis

cDOQ-related data retrieved from sequencing on Illumina platforms was first quality controlled using FastQC (15) and reads afterwards trimmed with Trimmomatic (16) to the first 40 nucleotides corresponding to the cDOQ hybridization sequence. Finally, trimmed reads were quantified using the Sailfish tool (17) under standard settings and a reference set including all cDOQ sequences (Supplementary Tables S2-S5). Analysis was performed using the European Galaxy Project platform (usegalaxy.eu) (18).

RNAseq data was first quality controlled using FastQC (15) and afterwards trimmed with Cutadapt (19), thus removing any remaining Illumina-related adapter sequences. Next, the trimmed reads were aligned to the reference sequences (total tRNA references from Modomics (12) and tRNAdb (11) using Bowtie 2 (20), accounting for the high modification prevalence in tRNA by allowing one mismatch within a 22 nt sequence. Finally, mapped reads were analyzed in the Integrative Genomics Viewer (21).

## Results

### Method design

For development of an accurate and sensitive high-throughput tRNA quantification method, we focused on reduction of known major bias sources, simple sample preparation, feasibility with equipment available in standard biochemical laboratories, and simple data workup.

**Figure 1.**
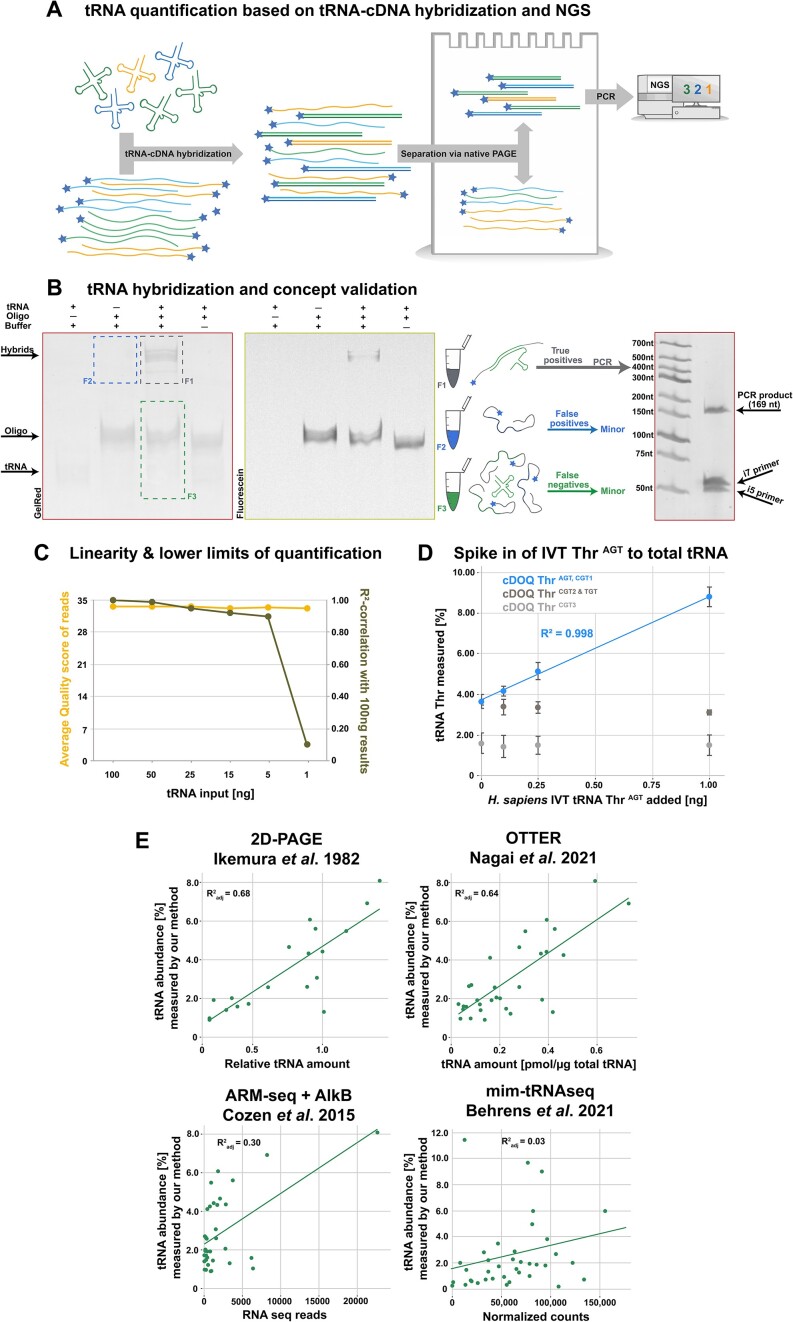
Overview of the hybridization-based quantification method and major performance characteristics. (**A**) Display of major steps in our hybridization-based quantification approach. (**B**) Example of hybridization on a native PAGE gel scanned for tagged fluorescein and GelRed staining. tRNA-cDOQ hybrids (F1) are physically separated from unbound oligo on the gel, afterwards excised and subjected to index PCR yielding full length product at 169 nt. Fraction F2 and F3 were analyzed in separate experiments (Supplementary Figure S2D-F) to characterize potential bias. (**C**) Average read quality scores yielded from samples prepared with decreasing amounts of tRNA displayed in yellow compared to *R*^2^-values of these samples correlated to a sample prepared from 100 ng tRNA. (**D**) Calibration and absolute quantification by standard addition of *in vitro* transcribed tRNA Thr ^AGT^ to 25 ng of total tRNA from HEK 293 cells plotted against the measured tRNA abundance. Biological triplicates, error bars indicate standard deviation. The y-axis interception corresponds to the tRNA abundance of the targeted isodecoders in the sample. (**E**) Correlation plots comparing *S. cerevisiae* tRNA abundances from our method with results obtained from other publications and *H. sapiens* for mim-tRNAseq.

At the core of the method is a hybridization step of the tRNA of interest with an engineered cDNA. This step transmits quantitative information from tRNA to the resulting tRNA-cDNA duplexes in a 1:1 stoichiometry. The duplexes are then physically separated from non-hybridized cDNA. This leaves the quantitative information in single-stranded cDNA from which it is easily read out. In contrast to e.g. RNAseq methods, this foregoes the use of numerous enzymatic conversion steps such as ligation and reverse transcription, thereby avoiding the associated biases. The only remaining enzymatic step is an index PCR for the subsequent quantification by NGS sequencing (Figure 1A).

In practice, an organism-specific mix of some 40 different cDNAs, each one corresponding to a major tRNA family, is hybridized to either total RNA or isolated total tRNA. Following denaturation and subsequent hybridization, the hybridization mixture is purified by native PAGE, thus physically separating tRNA-oligo hybrids from excess oligo and RNA contaminants. Upon elution from the native gel, a mixture of tRNA-oligo hybrids is obtained, in which the initial tRNA abundances are now represented by the abundance of each tRNA-specific oligo in the mixture. Quantification of the ∼40 sequences was here developed for Illumina NGS system but may conceivably be adapted to other analytical platforms. Conforming to this system, a bias-aware library preparation and analysis pipeline applies as follows. The oligo-containing gel eluate is directly subjected to low-cycle index PCR to minimize amplification bias. The resulting full-length product is amenable to sequencing on all current Illumina platforms and straightforward analysis requiring a minimum of training or prior knowledge. Data treatment comprises quality control, trimming and alignment. The final output is a quantitative table in which the sum of individual cDOQ reads divided by the total number of reads per sample represent the relative tRNA abundances in the initial RNA sample. Before application to several biological problems, this protocol was extensively scrutinized and validated with respect to various potential sources for errors and biases, as outlined below.

### Method characterization and validation

#### cDNA design integrates NGS adapters and tRNA hybridization sequences

Probe design for cDNA oligomers (abbreviated „cDOQ’ for cDNA Oligo for quantification) addressed the following criteria, namely (i) facile detection and visualization, (ii) sequence elements for subsequent quantification and (iii) quantitative hybridization of the cognate tRNA. A combination of cDOQs is needed to (iv) capture all known tRNA sequences of a given organism. Supplementary Figure S1A shows the general structure of cDOQs, comprising a 40 nt hybridization region complementary to the 3′end of a given tRNA isoacceptor family and framed by primer binding sites for the illumina i5 and i7 amplification sequences. The main rationale for choosing the 40 nt towards the 3′ end was sufficient sequence differentiation while maintaining acceptable cDOQ length. Also, the 3′modification landscape on e.g. human tRNAs comprises few Watson-Crick-impeding modifications, and this design allows separate targeting of 5′-tRFs (*vide infra*). The cDOQ construct is complemented by a 5′-fluorescent dye that aids visualization after PAGE. The process of cDOQ design considers all isodecoders and isoacceptors of a tRNA species, compiling them based on their levenshtein distance, a metric indicating the number of nucleotides different between two observed sequences, and thus sequence dissimilarity (Supplementary Figure S1B). Acknowledging the widespread use of acronyms in the field, we chose DORQ-seq for ‘DNA Oligo for RNA Quantification sequencing’.

#### A surprising PCR amplification bias is minimal at 6 replication cycles

When the composition of an equimolar mixture of ∼40 cDOQs, here directed against human tRNAs, was analyzed for relative content after 3, 6, 9 and 12 cycles of index PCR, a significant amplification bias was detected for 9 and 12 cycles. As shown in Supplementary Figure S1C, certain sequences were found enriched by 100% while others were decreased to 20% relative to the abundance at 2 cycles. This was unexpected given the identical primer binding sites and was only weakly correlated to the GC content of the hybridizing sequences (Supplementary Figure S1D). Interestingly, sequences behaved identically in three experimental replicates (executed on different days), demonstrating that the bias was indeed sequence specific and not random. Similar experiments on cDOQ mixtures covering tRNAs from other model organisms (Supplementary Figure S1E) further confirmed this, and consistently showed minimal bias after 6 cycles, which was henceforth the default setting.

#### cDOQ design is specific to single tRNA sequences

In an application to a first model organism, the 43 known cytosolic tRNA sequences of *Saccharomyces cerevisiae* were collapsed into 38 families, as described previously (14). Within the corresponding 38 cDOQs, the shortest Levenshtein distance was 6 nucleotides, occurring between Gly GCC and Gly CCC. This corresponds to 15.0% of the hybridizing sequence, slightly exceeding the recommendations of 10–12.5% (4–5 nt) by Letowski *et al.* (22). For experimental validation, 4 tRNAs were individually isolated from shifted bands in native gels after hybridization of total tRNA to their corresponding cDOQs. RNAseq of material isolated with the Gly GCC-cDOQ yielded 99% read alignment to the cognate tRNA Gly GCC family, and only 0.01% aligned to the nearest neighbor Gly CCC (Supplementary Figure S2A). Among the 4 tRNAs the average read mapping to the cognate tRNA sequence was 97% and the minimum 95% (Supplementary Figure S2B). Similar values were obtained with full-length cDOQs used for isolation of seven different tRNAs. This establishes a Levenshtein distance of 6 nucleotides as efficient in the discrimination of similar sequences by 2–4 orders of magnitude. The cDOQ-Gly GCC also caused near complete depletion of the cognate tRNA from the remaining i.e. unshifted tRNA material (Supplementary Figure S2C).

#### Transfer of quantitative information from tRNA to cDOQs

Moving from single sequences to total tRNA from *S. cerevisiae*, we performed an analogous experiment with a cocktail of 38 cDOQs. The mixture was heated and cooled to room temperature before separation on a native gel, which is shown in Figure 1B. The left panel images all nucleic acids as stained by gel red, while the right panel displays only the fluorescein-labeled cDOQs. The resulting tRNA:cDOQ heteroduplexes in lane 3 were well separated due to their retardation relative to tRNAs and cDOQs as evident by comparison with lanes 1 and 2. The duplexes were excised from the region designated by a black rectangle, eluted and further processed in our pipeline as ‘true positives’ (F1, black sample). The gel region corresponding to duplexes in lane 2 (F2 blue: ‘no tRNA’ false positive sample, blue rectangle in Figure 1B) was similarly treated to determine signals that might have arisen from nonstandard migration of non-hybridized cDOQs. A detailed analysis shown in Supplementary Figure S2D-E illustrated that the resulting signals in F2 were negligible. Additionally, the bottom part of lane 3 was excised, and putative residual RNA was submitted to an RNAseq protocol to potentially identify tRNAs that had failed to hybridize to the cDOQs (F3, green sample: ‘false negatives’). Its analysis, which is detailed in the supplement (Supplementary Figure S2F), involved reverse transcription of the RNA into cDNA, which was then submitted to short PCR-amplification and sequencing alongside the cDOQs. It showed only 0.05% of reads that could be mapped to cytosolic tRNA sequences, with the remainder corresponding to cDOQs, rRNA fragments and mitochondrial tRNA sequences. Finally, hybridization experiments using synthetic tRNA fragments (tRFs) displayed sufficient in-gel separation of mature tRNA species from tRFs, thus indicating a potential of this approach for tRNA fragment quantification and excluding tRFs as potential bias source for mature tRNA quantification (Supplementary Figure S2G). We thus concluded that all tRNA molecules were quantitatively hybridized and quantitative information on their composition was contained in the cDOQs of the F1 black sample.

#### Linearity to quantify the tRNA content of ∼2000 cells

To determine the lower limits of our method, a dilution series between 1 and 100 ng of *S. cerevisiae* tRNA was prepared and quantified. Figure 1C shows the average quality of reads and their similarity to the 100 ng composition plotted for each amount of input tRNA. Even from the lowest input of only 1 ng tRNA, excellent read quality could be achieved, with an average quality score of 33.8. Within the single-digit nanogram range of tRNA input, several ten-thousands of mapped reads, up to several million were achieved in our hands, depending on details of the sequencing platform and starting material. For all processed samples, read output proved sufficient, in theory, for relative quantification of the 38 species contained. However, the overall constant read quality, as well as greatly increased similarity to the 100 ng sample at 5 ng input and higher (Figure 1C), suggests an inherent limitation by noise. This places the lower limit for relative quantification around 5 ng tRNA input or slightly less, which, in our experiments, yielded 20000–100 000 mapped reads. This tRNA amount corresponds to the content of ∼30 000 yeast cells, or the equivalent of 2000 mammalian cells (23). Conceivably, with reduced sample number, adaptions in sequencing preparation and increased sequencing depth, high quality quantitative data might also be retrievable from lower tRNA amounts.

For absolute quantification, a spike-in calibration with an *in vitro* transcript of human tRNA^Thr AGT^ was quantified. The plot in Figure 1D shows an *R*^2^ value of 0.99 that linearly correlates read numbers with three amounts of IVT in 25 ng each of total human tRNA. The value for zero spike corresponds to ∼37 000 reads per million, equaling 0.93 ng of the targeted native tRNA Threonine isoacceptors. This comprises Thr AGT and Thr CGT-1, the latter an isodecoder with a single gene and presumably minor expression. Furthermore, this allows for absolute quantification of all other tRNAs analyzed within this sample, based on relative abundance. Additionally, similar experiments in *M. musculus* and prototype prokaryote *E. coli* displayed R2-correlation values of 0.99 and 0.97 respectively (Supplementary Figure S2H).

Finally, we compared our results to other published methods (Figure 1E), initially focusing on *S. cerevisiae* as widely used gold standard. Interestingly, we observed poor correlation with RNAseq based methods, but very good correlation with other hybridization-based methods, namely an *R*^2^_adj_ = 0.68 to 2D-gel electrophoresis results by Ikemura *et al.* (24) and an *R*^2^_adj_ of 0.64 with the recently published OTTER method by Nagai *et al.* (9), which utilizes fluorescent labeling and in-gel detection. In contrast, we found rather weak correlations when looking at quantitative RNAseq methods, e.g. an of *R*^2^_adj_ = 0.30 for ARMseq (25) data, and, now using HEK293T tRNA, an *R*^2^_adj_ of 0.03 (Supplementary Figure S2I) when comparing to mim-tRNAseq (8). Without being able to offer a molecular rationale, we conclude that hybridization-based methods on one hand, and RNAseq-based methods on the other hand cluster together.

#### Fast, precise, cost-effective and high-throughput quantification of tRNA

In exploiting the full throughput-capability of NGS, multiplexing was established for parallel analysis of up to 96 samples on any of the current Illumina platforms. A typical run on a Nextseq 2000 platform with 96 samples input yielded over 4 million reads per sample, with an average *Q*-Score of 33.6. This thus implies less than one error in 2200 base calls, meaning that fewer than 2% of the 40 nt recognition sequences was subject to a single base calling error mutation. Given the minimum Levenshtein distance of 6 between any two cDOQs, misassignments among cDOQs can be considered insignificant for quantification. Indeed, and in contrast to other methods (8,26) we have never observed any multi-mapping. Typically, >98% of the reads mapped to the 38 yeast cDOQ sequences, providing excellent statistics way above the experimental limit established above (Figure 1C). Of note, the data treatment is, in comparison to any other RNAseq approach, very simple and fast.

The turnaround time of such an analysis, starting from 96 samples of total RNA or total tRNA, is about 5 days, including 15–20 hours of hands-on time and 3–5 h of data analysis. Assuming that the NGS is outsourced, the overall cost including consumables and sequencing is about 50$ per sample. More importantly, this method evades all problems associated to reverse transcription, including the impact of RNA modifications, which are especially prevalent in tRNA. Consequently, implausible overrepresentation of certain tRNA species, known as ‘jackpot’ effects (7), and presumably arising from a low modification grade and consequently improved reverse transcription rate, do not occur, and the relative distribution of tRNA families is much more physiologically plausible. Because the method easily lends itself to numerous problems in tRNA biology, we have devised and validated species-specific sets of cDOQs for several model organisms including *E. coli*, mouse, and human, wherein we have also included mitochondrial tRNAs when appropriate (Supplementary Tables S1–S5). Among all organisms analyzed, the mapping rate ranged between 92 and 99% uniquely aligned reads (Supplementary Figure S2J).

#### Mitochondrial tRNA composition in mouse differs from human

**Figure 2.**
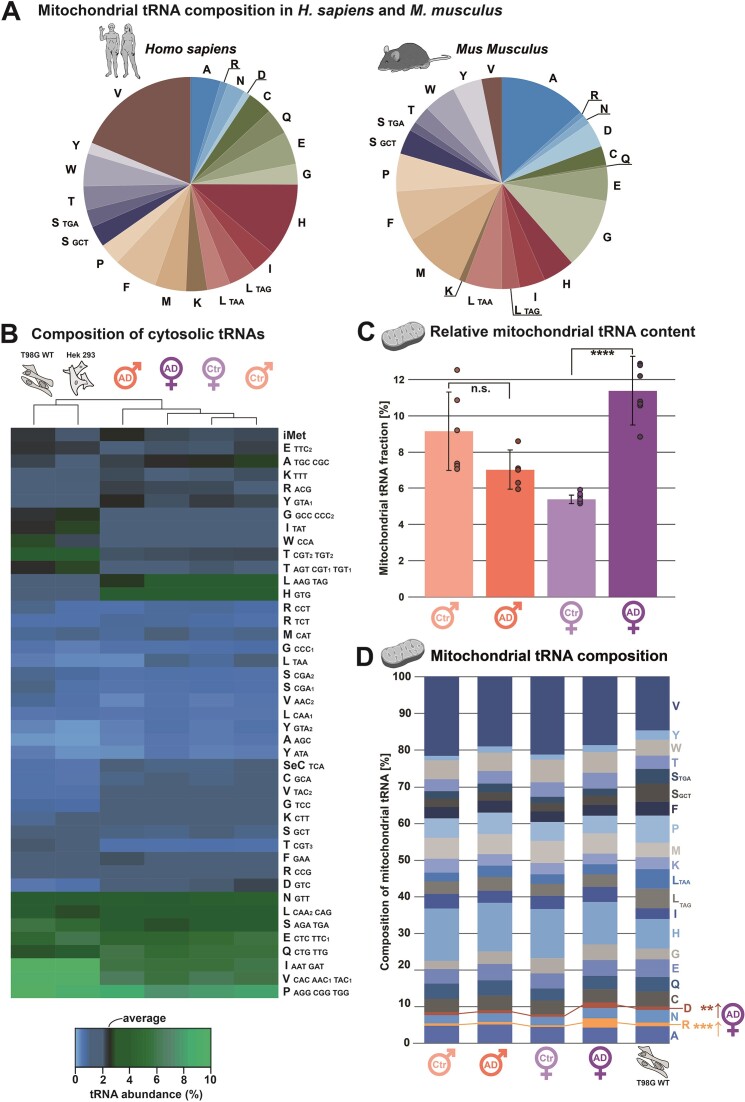
Analysis of tRNA expression in different mammals focusing on mitochondria and Alzheimer's disease. (**A**) Composition profile of all 22 mitochondrial tRNAs in *H. sapiens* and *M. musculus* from frontal cortex tissue. tRNAs are labeled in one-letter amino acid code with anticodons listed for isoacceptors if applicable. (**B**) Composition of 43 cytosolic tRNAs in two human cell lines and human cortex tissue from male and female individuals, either bearing Alzheimer's disease (AD) or being non-dement control (Ctr). The average abundance is visible in black with higher-than-average abundance in green and lesser abundance in blue. tRNAs are labeled in one-letter amino acid code with multiple anticodons listed when cDOQs target multiple isoacceptors at once. Anticodons are additionally numbered in cases where different isodecoders were targeted. Performed in technical triplicates. (**C**) Fraction of mitochondrial tRNA of all tRNAs analyzed in human cortex tissue from male and female individuals, either bearing Alzheimer's disease (AD) or being non-dement control (Ctr). Error bars indicate standard deviation and asterisk indicate a significant difference in one-sided *t*-test (**** *P* ≤ 0.0001) while n.s. indicates no significant difference. Performed in technical triplicates. (**D**) Display of mitochondrial tRNA composition in human cell line and human frontal cortex tissue from male and female individuals, either bearing Alzheimer's disease (AD) or being non-dement control (Ctr). Performed in technical triplicates, asteriske indicate a significant difference in one-sided *t*-test for the female AD group (** *P* ≤ 0.01, *** *P* ≤ 0.001).

An analysis covering both cytosolic and mitochondrial tRNA compositions was conducted in several human cell lines and tissues (vide infra). The mitochondrial tRNA composition consistently showed a ∼21% share of mt tRNA^Val^ (Figure 2A), reflecting the substitution of 5S rRNA in mammalian mitochondria by this tRNA (27,28), which further validates both the method and its application to mammals. Interestingly, the analogous analysis of mouse tissues from brain and liver (29) revealed only 3% abundance of mt-tRNA^Val^, while the most abundant mitochondrial species was now mt-tRNA^Ala^, featuring 13%, about 3× as much as in human. We were unable to find any literature on the identity of the 5S rRNA substitute in mouse, and, significantly, a paper summarizing the findings in five (30) mammalian species did not list mouse either. We conclude that an experimental analysis likely failed, and our data indicate that possibly the 5S rRNA role is filled by a mixture of different tRNA species in mouse.

#### Gender-specific variation of mitochondrial tRNA abundances in Alzheimer patients

In the context of Alzheimer's disease (AD), we applied our high-throughput quantification approach to a set of 25 human brain samples comprising 14 females and 11 males, both patients and healthy control. Additionally, several frequently used human cell lines were analyzed and compared to results from human tissue. Cytosolic tRNA abundance distributions showed no significant differences between patient groups, though clear differences when compared to human cell lines (Figure 2B). From 43 cDOQs targeting cytosolic tRNAs, 16 (HEK 293) or 21 (T98G WT) showed significant differences compared to human cortex tissue.

Strikingly, the overall abundance of mitochondrial tRNAs was significantly increased by 2-fold in women with AD, relative to the control group (Figure 2C). An increased level in male patients was not significant relative to the male control group. Investigating the relative abundances of individual mt-tRNAs, two significant differences could be observed for female patients. Both tRNA^ArgTCT^ and tRNA^AspGTC^ displayed significantly increased abundance compared to all other patient groups (Figure 2D). Interestingly, with the exception of the female AD group, no significant differences could be observed between cell lines and human samples on a mitochondrial level.

The increase in mitochondrial tRNAs in female Alzheimer patients is unexpected, yet not implausible. Mitochondrial function is a central focus in Alzheimer research (31,32), and it is also known that the molecular pathogenesis differs significantly between female and male patients. We therefore consider this data an incentive to develop quantification of tRNA in the AD field.

#### Severe differences in tRNA pool composition of *E. coli* cytosol and polysomes are further impacted by paraquat treatment

**Figure 3.**
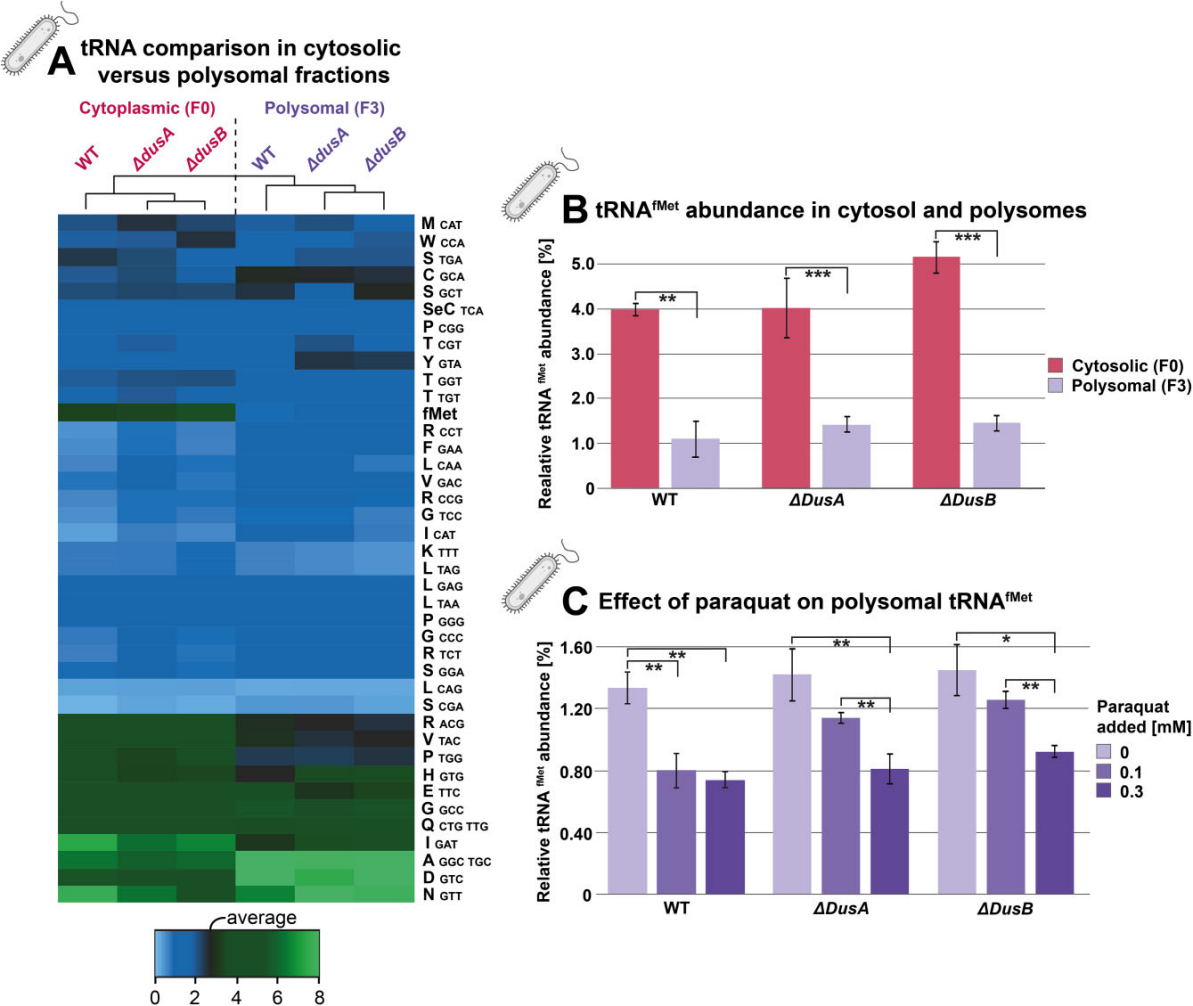
Differing composition of cytosolic and polysomal tRNA pools of *E. coli* mutants under oxidative stress. (**A**) Composition profile of 40 tRNAs in either cytoplasmic (F0) or polysomal (F3) fraction of polysome preparations from *E. coli* K12 wildtype and two mutant strains. tRNAs are labeled in one-letter amino acid code with anticodons listed for multiple isoacceptors if applicable. Asteriske indicate a significant difference (* *P* ≤ 0.05) in one sided t-test between F0 and F3 fraction of *ΔdusA* tRNA abundance. (**B**) tRNA ^fMet^ abundance in different fractions (F0/F3) for different mutant strains. Error bars indicate standard deviation and asterisk indicate a significant difference in one-sided t-test (** *P* ≤ 0.01, *** *P* ≤ 0.001). Performed in technical triplicates. (**C**) Relative abundance of tRNA ^fMet^ for different mutants in their respective polysomal fractions treated with different amounts of paraquat. Performed in technical triplicates. Error bars indicate standard deviation and asterisk indicate a significant difference in one-sided t-test (* *P* ≤ 0.05, ** *P* ≤ 0.01).

As an application that makes use of the low input requirements, *E. coli* tRNAs isolated from a density gradients (Supplementary Figure S3A and B) that separate actively translating polysomes (labeled F3 in Figure 3) from the cytoplasmic pool of tRNAs (F0) were compared. As little as 21 ng of isolated tRNA from F3 fractions was sufficient to prepare technical triplicates. In addition to a wildtype *E. coli K12* strain, we compared the tRNA composition in two knockout strains of tRNA modification enzymes DusA and DusB. The respective enzymes catalyze dihydrouridine modifications in the namesake D-domain of tRNAs (33,34) and might conceivably influence tRNA fitness and flexibility, possibly including accommodation on the ribosome. We also thought it possible that such an effect might be modulated upon treatment with paraquat, inducing oxidative stress and potentially altering both tRNA abundances and their usage on actively translating polysomes. A heatmap in Figure 3 gives a detailed view of the results. In summary, no general effects could be observed. Instead, abundances changed for single tRNA species, suggesting a tRNA-specific impact of dihydrouridination on its respective usage in translation. While no general influence on tRNA abundances was visible, pronounced differences were observed between all F0 and their respective F3 fractions, with up to 15 tRNAs significantly under- or overrepresented (Figure 3A). These included tRNA^fMet^, which was far less abundant in all polysomal (F3) fractions compared to the cytosol with differences up to 3.5 fold (Figure 3B), which is highly plausible given that this tRNA is expected to occur only in freshly initiating ribosomes, i.e. the 5′-most ribosome in each given polysome. F3 levels of tRNA^fMet^ were similar across mutant strains with only minor differences between F0 fractions. Interestingly, the tRNA^fMet^ populations in the F3 fractions decreased upon exposure to paraquat (Figure 3C). The latter was applied at sublethal concentrations to induce oxidative stress, leading to marked growth reduction, which is presumably related to the decreased population of initiator tRNA in translating polysomes.

## Discussion

Quantification methods for tRNA are a major driving force in tRNA biology, itself embedded in the larger field of protein translation. While tRNA biology has resurged along with RNA modification research, it has concomitantly become clear that tRNA modifications impose strong limitations to quantification. As a cardinal process in RNA quantification, cDNA hybridization to RNA can be designed to overcome negative effects from RNA modifications, typically by adjusting the cDNA length. Hence, when properly designed, cDNA-based quantification approaches, such as northern blotting, OTTER, MSTand RIP-chips, (see Supplementary Table S6) can avoid biases, albeit at the expense of sequence information. As in the above methods, our approach only reports information corresponding to the employed query sequences, thus missing modification information and details on isoacceptor composition within isoacceptor families covered by a common cDOQ. Among the previous, only chip-based methods tackle multiple sequences in a single experiment, which is otherwise the domain of deep sequencing approaches. However, RNAseq approaches have their intrinsic biases and statistical caveats in the quantification of complex mixtures, which are compounded in tRNA quantification, since modifications compound existing enzyme-based biases and introduce new ones. For example, reverse transcription is known to be hampered by RNA structure (35), which in turn is known to be reinforced by tRNA modifications. Reverse transcription arrest by modifications is known to affect quantification, albeit useful in the detection and quantification of those same modifications (36,37). Removal of a few selected modifications by enzymatic treatment e.g. with AlkB was introduced to improve cDNA synthesis and as a negative control to validate modification detection based on RT-arrest (25,38), yet introducing another enzymatic step prone to bias. Since, on a general level, incubation with any enzyme and the associate workup are not only time-consuming, put at risk additional RNA degradation and associated bias, we designed a streamlined process with a minimum of experimental steps and only a single enzymatic step. Ultimately, the enabling crucial step resides in the information transfer through hybridization of tRNAs with cDOQ, and subsequent separation. Interestingly, our results align well with other hybridization-based approaches, whereas RT-based approaches align amongst each other. It might arguably benefit the field to conduct a comparative study among labs using the various methods. The strengths of our new approach include very low material input requirements, low turnaround time, low cost and high throughput. While the method only returns information on sequences that are queried by cDOQs, disregarding all other sequences improves the signal-to-noise and leads to extremely simple bioinformatics. The method is thus competitive or leading in any of the above categories (see Supplementary Table S6), and its combined advantages make it possible to analyze complex experimental setups (typically 96 samples) with low abundant samples, e.g. tRNAs from polysomes or brain tissue in just one sequencing run. Furthermore, the method holds great potential considering an application to small RNAs such as microRNAs, siRNAs or any other small non-coding RNAs. Therefore, with further testing and target-dependent optimization, a wide application of our approach seems well feasible. In summary, the method takes tRNA quantification to an advanced level of practical application to low input and high samples numbers. As we have demonstrated with AD biopsies, the method is clearly suited for analysis of clinical samples.

## Supplementary Material

gkae765_Supplemental_Files

## Data Availability

The data underlying this article has been deposited in BioProject (https://www.ncbi.nlm.nih.gov/bioproject/) with the accession number PRJNA1088289.
