# Correction to ‘DORQ-seq: high-throughput quantification of femtomol tRNA pools by combination of cDNA hybridization and Deep sequencing’

**DOI:** 10.1093/nar/gkae900

**Published:** 2024-10-15

This is a correction to: Marco Kristen, Marc Lander, Lea-Marie Kilz, Lukas Gleue, Marko Jörg, Damien Bregeon, Djemel Hamdane, Virginie Marchand, Yuri Motorin, Kristina Friedland, Mark Helm, DORQ-seq: high-throughput quantification of femtomol tRNA pools by combination of cDNA hybridization and Deep sequencing, *Nucleic Acids Research*, 2024; gkae765, https://doi.org/10.1093/nar/gkae765

In the originally published version of this manuscript, the authors’ first names and surnames were erroneously transposed.

This has been corrected.

