# Supplementary material for: DORQ-seq: high-throughput quantification of femtomol tRNA pools by combination of cDNA hybridization and Deep sequencing
Source: Nucleic Acids Res. 2024 Sep 11;52(18):e89. doi: 10.1093/nar/gkae765 (PMC11472062; doi:10.1093/nar/gkae765)
Supplement: gkae765_Supplemental_Files [file gkae765_supplemental_files.zip › Kristen 2024 NAR supplement.docx]

**Supplementary Material**

**DORQ-seq: High-throughput quantification of femtomol tRNA pools by combination of cDNA hybridization and Deep Sequencing**

**AUTHORS**

Kristen Marco^1^, Lander Marc^1^, Kilz Lea-Marie^1^, Gleue Lukas^1^, Jörg Marko^1^, Bregeon Damien^2^, Hamdane Djemel ^3^, Marchand Virginie^4^, Motorin Yuri^4,5^, Friedland Kristina^1^, Helm Mark^1*^

^1^ Institute of Pharmaceutical and Biomedical Sciences, Johannes Gutenberg University Mainz, Staudingerweg 5, 55128, Mainz, Germany.

^2^ IBPS, Biology of Aging and Adaptation, Sorbonne Université, Paris 75252, France.

^3^ Laboratoire de Chimie des Processus Biologiques, CNRS-UMR 8229, Collège De France, Université Pierre et Marie Curie, 11 place Marcelin Berthelot, 75231 Paris, Cedex 05, France.

^4^ Université de Lorraine, IMoPA UMR7365 CNRS-UL, BioPole, 54000 Nancy, France.

^5^ Université de Lorraine, Epitranscriptomics and RNA Sequencing (EpiRNA-Seq) Core Facility, UAR2008 IBSLor (CNRS-UL)/US40 (INSERM), 54000 Nancy, France.

^*^Correspondence to mhelm@uni-mainz.de

Further characterization of the DORQ-seq method required analysis of potential noise derived from oligo secondary structures and the completeness of hybridization (**Fig. S2 D**). In order to determine potential noise, human tRNA-oligo hybrids (F1) of different human total RNA samples were excised from a native PAGE gel, as well as the corresponding height for samples only bearing oligo (F2), thus excising potential oligo running at hybrid-size. Following elution, 50 fmol of *E. coli* Phe ^GAA^ hybridization oligo were added to each sample as deliberate contaminant and internal standard. Following index PCR and Illumina sequencing, the percentage of reads mapped to *E. coli* Phe ^GAA^ (corresponding to 50 fmol oligo) was used to calculate the initial amount of *H. sapiens* hybridization oligos before PCR. Calculated oligo amounts ranged between 2.8 ng and 14.7 ng for hybrid (F1) fractions compared to 0.5 ng for the F2 fraction. Correlation analysis of retrieved data (**Fig. S2 E**) compared the individual measured tRNA abundances with the average tRNA abundances calculated from all hybrid (F1) fractions. Adjusted R^2^-values ranged between 0.94 and 0.98 for F1 fractions and 0.4 for the potential noise, thus indicating great differences in noise composition compared to hybrids. No dependency between R^2^-value and initial *H. sapiens* oligo amount could be observed as correlation was constantly high across all F1 fractions, implying a very limited impact of potential noise.


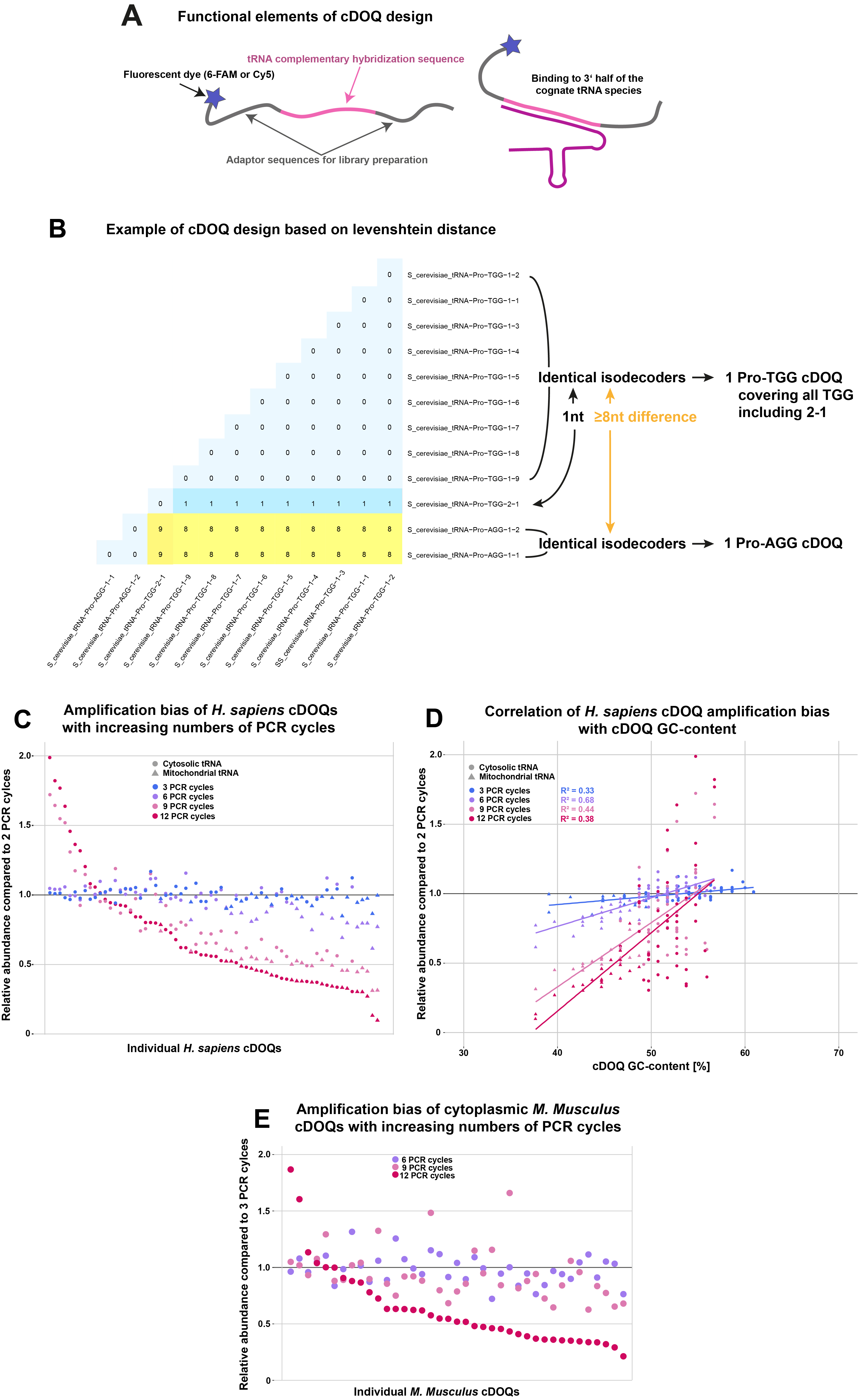


**Supplementary Figure 1: cDOQ structure and PCR-derived bias in cDOQ quantities. A** General structure of a cDNA oligo for quantification (”cDOQ”) and its binding to the cognate tRNA **B** Design approach for cDOQs as shown for S.cerevisiae tRNA Proline. The levenshtein distance of all known isoacceptors and isodecoders is shown in the heatmap with numbers indicating the levenshtein distance between two sequences. Sequences with less than 6nt distance will be grouped as one cDOQ for the respective isodecoders or, if applicable, even isoacceptors. **C** Relative abundance of individual cDOQs in a mixture of *H. sapiens* cDOQs after different numbers of PCR cycles compared to the initial abundance measured after 2 PCR cycles. Data shape indicates cDOQs targeting cytosolic (circle) or mitochondrial (triangle) tRNAs. **D** Correlation the cDOQ GC-content with the relative abundance of individual cDOQs in a mixture of *H. sapiens* cDOQs after different numbers of PCR cycles compared to the initial abundance measured after 2 PCR cycles. Data shape indicates cDOQs targeting cytosolic (circle) or mitochondrial (triangle) tRNAs. **E** Relative abundance of individual cDOQs in a mixture of *M. musculus* cDOQs after different numbers of PCR cycles compared to the initial abundance measured after 3 PCR cycles.


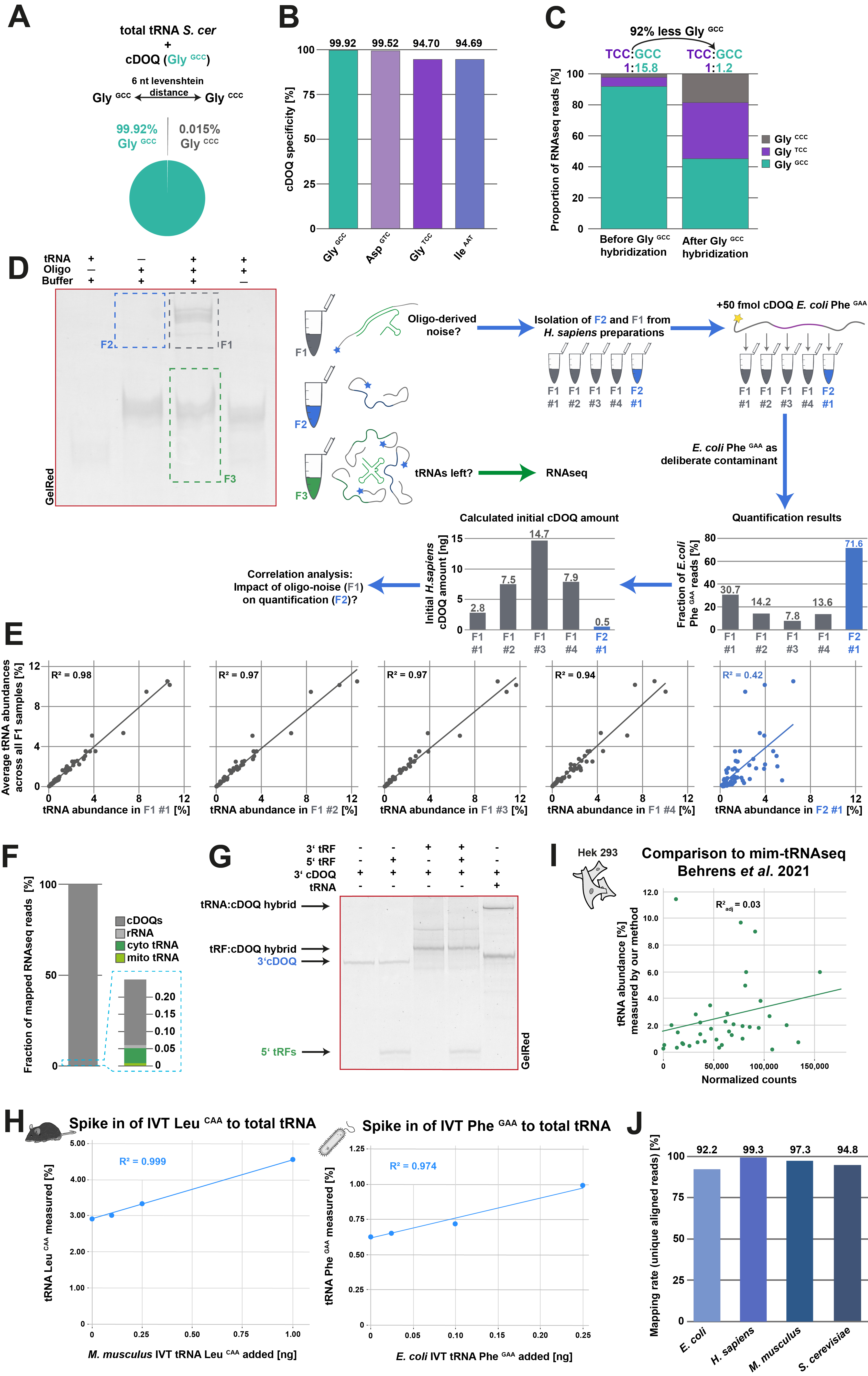


**Supplementary Figure 2: Investigation of oligo specificity, potential quantitative bias and completeness of hybridization. A** Percentage of reads mapped to either glycine tRNA isoacceptor after specifically targeting Gly ^GCC^ with the respective cDOQ. The isoacceptors display 6 nt difference in the hybridization sequence (6 nt = 15%). **B** Exemplary cDOQ specificity. **C** Ratio of RNAseq reads from different tRNA Gly isoacceptors before and after specific hybridization of tRNA Gly ^GCC^. **D** In the process of hybridization, the noise derived from oligo secondary structures (F2) and its respective impact on hybrids (F1) and following quantification was investigated. Furthermore, completeness of hybridization was analyzed by RNAseq analysis of tRNA-height post-hybridization (F3). 12 fractions were prepared from four human total RNA samples with F2 running on the same hybridization gel. Following elution, 50 fmol of hybridization oligo targeting *E. coli* Phe ^GAA^ was added to all F1 and F2 fractions. Samples were subjected to PCR, sequenced and afterwards analyzed for their *E. coli* Phe ^GAA^ oligo read proportion. From this, the initial oligo concentration could be calculated for each sample and used for impact analysis. **E** Correlation plots for all F1 and F2 samples comparing tRNA abundance in the respective sample with the average abundance across all four F1 fractions. **F** Comparison of mapped reads retrieved for cDOQs and RNA species from RNAseq analysis of Fraction 3 (F3). **G**Hybridization and separation of tRNA fragments and mature tRNAs *via* cDOQs on a PAGE. **H**Standard addition experiments adding in vitro transcribed tRNA Isoacceptors to total tRNA of the respective species. **I** Correlation of tRNA quantification results for Hek293 cells obtained via cDOQs and mim-tRNAseq. **J** Mapping rate of uniquely aligned reads with the novel quantification approach across different

species **
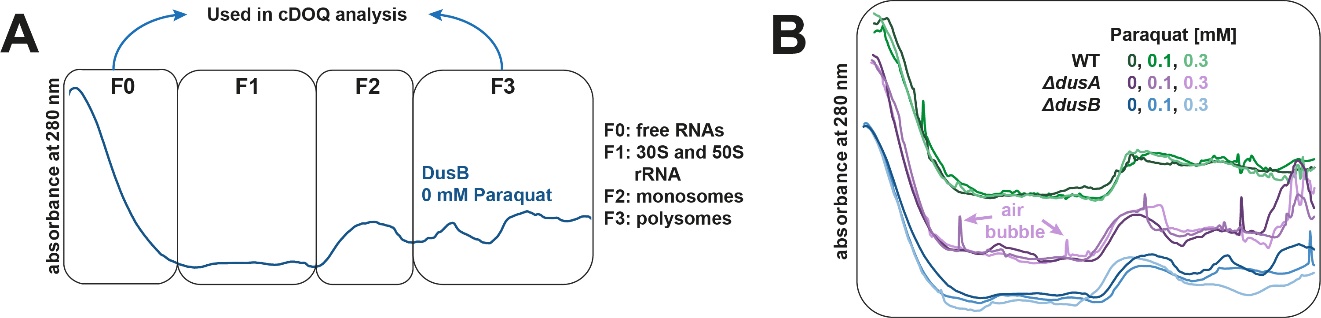
**

**Supplementary Figure 3: Preparation of polysome fractions to characterize tRNA pools involved In translation. A** Exemplary gradient curve from polysome preparation, indicating the fractions used in cDOQ-analysis. **B** Overview of all polysome profiles generated for *E. coli* WT (K12), *ΔdusA* and *ΔdusB* strains under treatment with increasing concentrations of paraquat.
