# Supplementary material for: DORQ-seq: high-throughput quantification of femtomol tRNA pools by combination of cDNA hybridization and Deep sequencing
Source: Nucleic Acids Res. 2024 Sep 11;52(18):e89. doi: 10.1093/nar/gkae765 (PMC11472062; doi:10.1093/nar/gkae765)
Supplement: gkae765_Supplemental_Files [file gkae765_supplemental_files.zip › Supplementary_Tables_Kristen_et_al.docx]

**Supplementary Table 1: Custom read and indexing primers**

| **Primer** | **Sequence** |
| --- | --- |
| i5 index read | 5'-GATCGTCGGACTGTAGAACTCTGAAC-3' |
| i 501 | 5'-AATGATACGGCGACCACCGAGATCTACACTATAGCCTGTTCAGAGTTCTACAGTCCGACGATC-3' |
| i 502 | 5'-AATGATACGGCGACCACCGAGATCTACACATAGAGGCGTTCAGAGTTCTACAGTCCGACGATC-3' |
| i 503 | 5'-AATGATACGGCGACCACCGAGATCTACACCCTATCCTGTTCAGAGTTCTACAGTCCGACGATC-3' |
| i 504 | 5'-AATGATACGGCGACCACCGAGATCTACACGGCTCTGAGTTCAGAGTTCTACAGTCCGACGATC-3' |
| i 505 | 5'-AATGATACGGCGACCACCGAGATCTACACAGGCGAAGGTTCAGAGTTCTACAGTCCGACGATC-3' |
| i 506 | 5'-AATGATACGGCGACCACCGAGATCTACACTAATCTTAGTTCAGAGTTCTACAGTCCGACGATC-3' |
| i 507 | 5'-AATGATACGGCGACCACCGAGATCTACACACGTCCTGGTTCAGAGTTCTACAGTCCGACGATC-3' |
| i 508 | 5'-AATGATACGGCGACCACCGAGATCTACACGTACTGACGTTCAGAGTTCTACAGTCCGACGATC-3' |
| i 701 | 5'-CAAGCAGAAGACGGCATACGAGATCGAGTAATGTGACTGGAGTTCAGACGTGTGCTCTTCCGATCT-3' |
| i 702 | 5'-CAAGCAGAAGACGGCATACGAGATTCTCCGGAGTGACTGGAGTTCAGACGTGTGCTCTTCCGATCT-3' |
| i 703 | 5'-CAAGCAGAAGACGGCATACGAGATAATGAGCGGTGACTGGAGTTCAGACGTGTGCTCTTCCGATCT-3' |
| i 704 | 5'-CAAGCAGAAGACGGCATACGAGATGGAATCTCGTGACTGGAGTTCAGACGTGTGCTCTTCCGATCT-3' |
| i 705 | 5'-CAAGCAGAAGACGGCATACGAGATTTCTGAATGTGACTGGAGTTCAGACGTGTGCTCTTCCGATCT-3' |
| i 706 | 5'-CAAGCAGAAGACGGCATACGAGATACGAATTCGTGACTGGAGTTCAGACGTGTGCTCTTCCGATCT-3' |
| i 707 | 5'-CAAGCAGAAGACGGCATACGAGATAGCTTCAGGTGACTGGAGTTCAGACGTGTGCTCTTCCGATCT-3' |
| i 708 | 5'-CAAGCAGAAGACGGCATACGAGATGCGCATTAGTGACTGGAGTTCAGACGTGTGCTCTTCCGATCT-3' |
| i 709 | 5'-CAAGCAGAAGACGGCATACGAGATCATAGCCGGTGACTGGAGTTCAGACGTGTGCTCTTCCGATCT-3' |
| i 710 | 5'-CAAGCAGAAGACGGCATACGAGATTTCGCGGAGTGACTGGAGTTCAGACGTGTGCTCTTCCGATCT-3' |
| i 711 | 5'-CAAGCAGAAGACGGCATACGAGATGCGCGAGAGTGACTGGAGTTCAGACGTGTGCTCTTCCGATCT-3' |
| i 712 | 5'-CAAGCAGAAGACGGCATACGAGATCTATCGCTGTGACTGGAGTTCAGACGTGTGCTCTTCCGATCT-3' |

**Supplementary Table 2: cDOQs designed for *E. coli***

| *Escherichia coli* | **tRNA** | **Anticodon** | **Sequence** |
| --- | --- | --- | --- |
|  | Ala | GGC & TGC | 5‘-AGACGTGTGCTCTTCCGATCTTGGTGGAGCTAAGCGGGATCGAACCGCAGACCTCCTGCATGATCGTCGGACTGTAGAACTCTGAAC-3’ |
|  | Arg | ACG | 5‘-AGACGTGTGCTCTTCCGATCTTGGTGCATCCGGGAGGATTCGAACCTCCGACCGCTCGGTTGATCGTCGGACTGTAGAACTCTGAAC-3’ |
|  | Arg | CCG | 5‘-AGACGTGTGCTCTTCCGATCTTGGCGCGCCCGACAGGATTCGAACCTGAGACCTCTGCCTCGATCGTCGGACTGTAGAACTCTGAAC-3’ |
|  | Arg | CCT | 5‘-AGACGTGTGCTCTTCCGATCTTGGTGTCCCCTGCAGGAATCGAACCTGCAATTAGCCCTTAGATCGTCGGACTGTAGAACTCTGAAC-3’ |
|  | Arg | TCT | 5‘-AGACGTGTGCTCTTCCGATCTTGGCGCGCCCTGCAGGATTCGAACCTGCGGCCCACGACTTGATCGTCGGACTGTAGAACTCTGAAC-3’ |
|  | Asn | GTT | 5‘-AGACGTGTGCTCTTCCGATCTTGGCTCCTCTGACTGGACTCGAACCAGTGACATACGGATTGATCGTCGGACTGTAGAACTCTGAAC-3’ |
|  | Asp | GTC | 5‘-AGACGTGTGCTCTTCCGATCTTGGCGGAACGGACGGGACTCGAACCCGCGACCCCCTGCGTGATCGTCGGACTGTAGAACTCTGAAC-3’ |
|  | Cys | GCA | 5‘-AGACGTGTGCTCTTCCGATCTTGGAGGCGCGTTCCGGAGTCGAACCGGACTAGACGGATTTGATCGTCGGACTGTAGAACTCTGAAC-3’ |
|  | fMET | CAT | 5‘-AGACGTGTGCTCTTCCGATCTTGGTTGCGGGGGCCGGATTTGAACCGACGACCTTCGGGTTGATCGTCGGACTGTAGAACTCTGAAC-3’ |
|  | Gln | CTG & TTG | 5‘-AGACGTGTGCTCTTCCGATCTTGGCTGGGGTACCTGGATTCGAACCTCGGAATGCCGGAATGATCGTCGGACTGTAGAACTCTGAAC-3’ |
|  | Glu | TTC | 5‘-AGACGTGTGCTCTTCCGATCTTGGCGTCCCCTAGGGGATTCGAACCCCTGTTACCGCCGTGGATCGTCGGACTGTAGAACTCTGAAC-3’ |
|  | Gly | GCC | 5‘-AGACGTGTGCTCTTCCGATCTTGGAGCGGGAAACGAGACTCGAACTCGCGACCCCGACCTTGATCGTCGGACTGTAGAACTCTGAAC-3’ |
|  | Gly | CCC | 5‘-AGACGTGTGCTCTTCCGATCTTGGAGCGGGCGAAGGGAATCGAACCCTCGTATAGAGCTTGGATCGTCGGACTGTAGAACTCTGAAC-3’ |
|  | Gly | TCC | 5‘-AGACGTGTGCTCTTCCGATCTTGGAGCGGGCAGCGGGAATCGAACCCGCATCATCAGCTTGGATCGTCGGACTGTAGAACTCTGAAC-3’ |
|  | His | GTG | 5‘-AGACGTGTGCTCTTCCGATCTTGGGGTGGCTAATGGGATTCGAACCCACGACAACTGGAATGATCGTCGGACTGTAGAACTCTGAAC-3’ |
|  | Ile | GAT | 5‘-AGACGTGTGCTCTTCCGATCTTGGTAGGCCTGAGTGGACTTGAACCACCGACCTCACCCTTGATCGTCGGACTGTAGAACTCTGAAC-3’ |
|  | Ile | CAT | 5‘-AGACGTGTGCTCTTCCGATCTTGGTGGCCCTTGCTGGACTTGAACCAGCGACCAAGCGATTGATCGTCGGACTGTAGAACTCTGAAC-3’ |
|  | Leu | CAG | 5‘-AGACGTGTGCTCTTCCGATCTTGGTGCGAGGGGGGGGACTTGAACCCCCACGTCCGTAAGGGATCGTCGGACTGTAGAACTCTGAAC-3’ |
|  | Leu | CAA | 5‘-AGACGTGTGCTCTTCCGATCTTGGTGCCGAAGGCCGGACTCGAACCGGCACGTATTTCTACGATCGTCGGACTGTAGAACTCTGAAC-3’ |
|  | Leu | GAG | 5‘-AGACGTGTGCTCTTCCGATCTTGGTACCGAGGACGGGACTTGAACCCGTAAGCCCTATTGGGATCGTCGGACTGTAGAACTCTGAAC-3’ |
|  | Leu | TAA | 5‘-AGACGTGTGCTCTTCCGATCTTGGTACCCGGAGCGGGACTTGAACCCGCACAGCGCGAACGGATCGTCGGACTGTAGAACTCTGAAC-3’ |
|  | Leu | TAG | 5‘-AGACGTGTGCTCTTCCGATCTTGGTGCGGGAGGCGAGACTTGAACTCGCACACCTTGCGGCGATCGTCGGACTGTAGAACTCTGAAC-3’ |
|  | Lys | TTT | 5‘-AGACGTGTGCTCTTCCGATCTTGGTGGGTCGTGCAGGATTCGAACCTGCGACCAATTGATTGATCGTCGGACTGTAGAACTCTGAAC-3’ |
|  | Met | CAT | 5‘-AGACGTGTGCTCTTCCGATCTTGGTGGCTACGACGGGATTCGAACCTGTGACCCCATCATTGATCGTCGGACTGTAGAACTCTGAAC-3’ |
|  | Phe | GAA | 5‘-AGACGTGTGCTCTTCCGATCTTGGTGCCCGGACTCGGAATCGAACCAAGGACACGGGGATTGATCGTCGGACTGTAGAACTCTGAAC-3’ |
|  | Pro | CGG | 5‘-AGACGTGTGCTCTTCCGATCTTGGTCGGTGATAGAGGATTCGAACCTCCGACCCCTTCGTCGATCGTCGGACTGTAGAACTCTGAAC-3’ |
|  | Pro | GGG | 5‘-AGACGTGTGCTCTTCCGATCTTGGTCGGCACGAGAGGATTTGAACCTCCGACCCCCGACACGATCGTCGGACTGTAGAACTCTGAAC-3’ |
|  | Pro | TGG | 5‘-AGACGTGTGCTCTTCCGATCTTGGTCGGCGAGAGAGGATTCGAACCTCCGACCCACTGGTCGATCGTCGGACTGTAGAACTCTGAAC-3’ |
|  | Sec | TCA | 5‘-AGACGTGTGCTCTTCCGATCTTGGCGGAAGATCACAGGAGTCGAACCTGCCCGGGACCGCTGATCGTCGGACTGTAGAACTCTGAAC-3’ |
|  | Ser | GGA | 5‘-AGACGTGTGCTCTTCCGATCTTGGCGGTGAGGGGGGGATTCGAACCCCCGATACGTTGCCGGATCGTCGGACTGTAGAACTCTGAAC-3’ |
|  | Ser | CGA | 5‘-AGACGTGTGCTCTTCCGATCTTGGCGGAGAGAGGGGGATTTGAACCCCCGGTAGAGTTGCCGATCGTCGGACTGTAGAACTCTGAAC-3’ |
|  | Ser | GCT | 5‘-AGACGTGTGCTCTTCCGATCTTGGCGGTGAGGCGGGGATTCGAACCCCGGATGCAGCTTTTGATCGTCGGACTGTAGAACTCTGAAC-3’ |
|  | Ser | TGA | 5‘-AGACGTGTGCTCTTCCGATCTTGGCGGAAGCGCAGAGATTCGAACTCTGGAACCCTTTCGGGATCGTCGGACTGTAGAACTCTGAAC-3’ |
|  | Thr | CGT | 5‘-AGACGTGTGCTCTTCCGATCTTGGTGCCGATAATAGGAGTCGAACCTACGACCTTCGCATTGATCGTCGGACTGTAGAACTCTGAAC-3’ |
|  | Thr | GGT | 5‘-AGACGTGTGCTCTTCCGATCTTGGTGCTGATACCCAGAGTCGAACTGCCGACCTCACCCTTGATCGTCGGACTGTAGAACTCTGAAC-3’ |
|  | Thr | TGT | 5‘-AGACGTGTGCTCTTCCGATCTTGGTGCCGACTACCGGAATCGAACTGGTGACCTACTGATTGATCGTCGGACTGTAGAACTCTGAAC-3’ |
|  | Trp | CCA | 5‘-AGACGTGTGCTCTTCCGATCTTGGCAGGGGCGGAGAGACTCGAACTCCCAACACCCGGTTTGATCGTCGGACTGTAGAACTCTGAAC-3’ |
|  | Tyr | GTA | 5‘-AGACGTGTGCTCTTCCGATCTTGGTGGTGGGGGAAGGATTCGAACCTTCGAAGTCGATGACGATCGTCGGACTGTAGAACTCTGAAC-3’ |
|  | Val | TAC | 5‘-AGACGTGTGCTCTTCCGATCTTGGTGGGTGATGACGGGATCGAACCGCCGACCCCCTCCTTGATCGTCGGACTGTAGAACTCTGAAC-3’ |
|  | Val | GAC | 5‘-AGACGTGTGCTCTTCCGATCTTGGTGCGTCCGAGTGGACTCGAACCAACGACCCCCACCATGATCGTCGGACTGTAGAACTCTGAAC-3’ |

**Supplementary Table 3: cDOQs designed for *S. cerevisiae***

| *Saccharomyces cerevisiae* | **Type** | **tRNA** | **Anticodon** | **Sequence** |
| --- | --- | --- | --- | --- |
|  | mitochondrial | Ala | TGC | 5‘-AGACGTGTGCTCTTCCGATCTTGGTGGAGTTAATGAGACTTGAACTCATATTAAATGCATGGATCGTCGGACTGTAGAACTCTGAAC-3‘ |
|  |  | Arg | ACG | 5‘-AGACGTGTGCTCTTCCGATCTTGGAATATCTTATAGGATTTGAACCTATATAATTAGATTCGATCGTCGGACTGTAGAACTCTGAAC-3‘ |
|  |  | Arg | TCT | 5‘-AGACGTGTGCTCTTCCGATCTTGGTACTCTCTCCATGATTTGAACATGGAATATTAATATTGATCGTCGGACTGTAGAACTCTGAAC-3‘ |
|  |  | Asn | GTT | 5‘-AGACGTGTGCTCTTCCGATCTTGGCGTCCTTAATAGGAATTGAACCCATCTATTCTCATTAGATCGTCGGACTGTAGAACTCTGAAC-3‘ |
|  |  | Asp | GTC | 5‘-AGACGTGTGCTCTTCCGATCTTGGCGAATCTAATCAGATTTGCACTGACATCCTCCATTATGATCGTCGGACTGTAGAACTCTGAAC-3‘ |
|  |  | Cys | GCA | 5‘-AGACGTGTGCTCTTCCGATCTTGGAAGAGATGAAGAGAATCGAACTCTTAATAAGTAGATTGATCGTCGGACTGTAGAACTCTGAAC-3‘ |
|  |  | iMet | CAT | 5‘-AGACGTGTGCTCTTCCGATCTTGGTAGCAATAATACGATTTGAACGTATATAATTAGGTCAGATCGTCGGACTGTAGAACTCTGAAC-3‘ |
|  |  | Gln | TTG | 5‘-AGACGTGTGCTCTTCCGATCTTGGTTGAATCGCTTTGATTCGAACAAAGAACTCCAAACTCGATCGTCGGACTGTAGAACTCTGAAC-3‘ |
|  |  | Glu | TTC | 5‘-AGACGTGTGCTCTTCCGATCTTGGTAACCTTAATCGGAATCGAACCGATATTATCAACATGGATCGTCGGACTGTAGAACTCTGAAC-3‘ |
|  |  | Gly | TCC | 5‘-AGACGTGTGCTCTTCCGATCTTGGTATAGATAGCGAGAATCGAACTCGCATTCAATGTTTGGATCGTCGGACTGTAGAACTCTGAAC-3‘ |
|  |  | His | GTG | 5‘-AGACGTGTGCTCTTCCGATCTTGGGGTGAATACTGAGAATCGAACTCAGATTTAACGCACCGATCGTCGGACTGTAGAACTCTGAAC-3‘ |
|  |  | Ile | GAT | 5‘-AGACGTGTGCTCTTCCGATCTTGGTGAAACTAACAGGGATTGAACCTATATTTGTACCTTAGATCGTCGGACTGTAGAACTCTGAAC-3‘ |
|  |  | Leu | TAA | 5‘-AGACGTGTGCTCTTCCGATCTTGGTGCTATTTAAAGGACTTGAACCTTCATACTGTAAAGTGATCGTCGGACTGTAGAACTCTGAAC-3‘ |
|  |  | Lys | TTT | 5‘-AGACGTGTGCTCTTCCGATCTTGGTGAGAATAGCTGGAGTTGAACCTGCATGGGTTGCTTAGATCGTCGGACTGTAGAACTCTGAAC-3‘ |
|  |  | Met | CAT | 5‘-AGACGTGTGCTCTTCCGATCTTGGTACTTGTAGAAGGAATTGAACCTTACATTATTTATTTGATCGTCGGACTGTAGAACTCTGAAC-3‘ |
|  |  | Phe | GAA | 5‘-AGACGTGTGCTCTTCCGATCTTGGTGCCCTTAATGAGAATCGAACTTATGTAAATAAATCTGATCGTCGGACTGTAGAACTCTGAAC-3‘ |
|  |  | Pro | TGG | 5‘-AGACGTGTGCTCTTCCGATCTTGGTCAGATAGGATAGACTCGAACTAACTAGGTCTTTCTCGATCGTCGGACTGTAGAACTCTGAAC-3‘ |
|  |  | Ser | GCT | 5‘-AGACGTGTGCTCTTCCGATCTTGGCGGAAAATATGAGATTCGAACTCATAAGAATTTACAAGATCGTCGGACTGTAGAACTCTGAAC-3‘ |
|  |  | Ser | TGA | 5‘-AGACGTGTGCTCTTCCGATCTTGGCGGATGATGTAGGATTTGAACCTACGTAGCCAATAAAGATCGTCGGACTGTAGAACTCTGAAC-3‘ |
|  |  | Thr | TAG | 5‘-AGACGTGTGCTCTTCCGATCTTGGTGTAAATACTAAGATTTGAACTTAGATAATATGCACCGATCGTCGGACTGTAGAACTCTGAAC-3‘ |
|  |  | Thr | TGT | 5‘-AGACGTGTGCTCTTCCGATCTTGGTGTTATATTAGGGATTTGAACCCCAAACCTTTCGATTGATCGTCGGACTGTAGAACTCTGAAC-3‘ |
|  |  | Trp | TCA | 5‘-AGACGTGTGCTCTTCCGATCTTGGCAAGGATAAAGAGATTCGAACTCCTAATGATTGATTTGATCGTCGGACTGTAGAACTCTGAAC-3‘ |
|  |  | Tyr | GTA | 5‘-AGACGTGTGCTCTTCCGATCTTGGTAAAGGGAATAGGAATTGAACCTATGAAGACCTAAGTGATCGTCGGACTGTAGAACTCTGAAC-3‘ |
|  |  | Val | TAC | 5‘-AGACGTGTGCTCTTCCGATCTTGGTAGGAAATATAGGGTTCGAACCTATAATCTTTCGTGTGATCGTCGGACTGTAGAACTCTGAAC-3‘ |
|  | cytosolic | Ala | AGC | 5‘-AGACGTGTGCTCTTCCGATCTTGGTGGACGAGTCCGGAATCGAACCGGAGACCTCTCCCACGATCGTCGGACTGTAGAACTCTGAAC-3‘ |
|  |  | Ala | TGC | 5‘-AGACGTGTGCTCTTCCGATCTTGGGTGGACGCAACCGGAATCGAACCGATGACCTCTTCCTGATCGTCGGACTGTAGAACTCTGAAC-3‘ |
|  |  | Arg | TCT | 5‘-AGACGTGTGCTCTTCCGATCTTGGCACTCACGATGGGGGTCGAACCCATAATCTTCTGATTGATCGTCGGACTGTAGAACTCTGAAC-3‘ |
|  |  | Arg | CCG | 5‘-AGACGTGTGCTCTTCCGATCTTGGAGCTCCTCCCGGGACTCGAACCCGGATCACAGCCACCGATCGTCGGACTGTAGAACTCTGAAC-3‘ |
|  |  | Arg | CCT | 5‘-AGACGTGTGCTCTTCCGATCTTGGCGTTCCGTACGGGACTCGAACCCGCAGTCTTCTCCTTGATCGTCGGACTGTAGAACTCTGAAC-3‘ |
|  |  | Arg | ACG | 5‘-AGACGTGTGCTCTTCCGATCTTGGCTTCCCCGCCAGGACTTGAACCTGGAATCTTCTGGTTGATCGTCGGACTGTAGAACTCTGAAC-3‘ |
|  |  | Asn | GTT | 5‘-AGACGTGTGCTCTTCCGATCTTGGCGACCCCAGTGAGGGTTGAACTCACGATCTTGCGATTGATCGTCGGACTGTAGAACTCTGAAC-3‘ |
|  |  | Asp | GTC | 5‘-AGACGTGTGCTCTTCCGATCTTGGCTCCGCGACGGGGAATTGAACCCCGATCTGGCACGCGGATCGTCGGACTGTAGAACTCTGAAC-3‘ |
|  |  | Cys | GCA | 5‘-AGACGTGTGCTCTTCCGATCTTGGAGCTCGCACTCAGGATCGAACTAAGGACCAACAGATTGATCGTCGGACTGTAGAACTCTGAAC-3‘ |
|  |  | iMet | CAT | 5‘-AGACGTGTGCTCTTCCGATCTTGGTAGCGCCGCTCGGTTTCGATCCGAGGACATCAGGGTTGATCGTCGGACTGTAGAACTCTGAAC-3‘ |
|  |  | Gln | TTG & CTG | 5‘-AGACGTGTGCTCTTCCGATCTTGGTCTTACCCGGATTCGAACCGGGGTTGTTCGGATCAAAGATCGTCGGACTGTAGAACTCTGAAC-3‘ |
|  |  | Glu | CTC & TTC | 5‘-AGACGTGTGCTCTTCCGATCTTGGCTCCGATACGGGGAGTCGAACCCCGGTCTCCACGGTGGATCGTCGGACTGTAGAACTCTGAAC-3‘ |
|  |  | Gly | TCC | 5‘-AGACGTGTGCTCTTCCGATCTTGGTGAGCGGTACGAGAATCGAACCCGTGTCCCCACCTTGGATCGTCGGACTGTAGAACTCTGAAC-3‘ |
|  |  | Gly | CCC | 5‘-AGACGTGTGCTCTTCCGATCTTGGTGCGGAAGCCGGGAATCGAACCCGGGCCCCATGCTTGGATCGTCGGACTGTAGAACTCTGAAC-3‘ |
|  |  | Gly | GCC | 5‘-AGACGTGTGCTCTTCCGATCTTGGTGCGCAAGCCCGGAATCGAACCGGGGGCCCAACGATGGATCGTCGGACTGTAGAACTCTGAAC-3‘ |

| *Saccharomyces cerevisiae* | **Type** | **tRNA** | **Anticodon** | **Sequence** |
| --- | --- | --- | --- | --- |
|  | cytosolic | His | GTG* | 5‘-AGACGTGTGCTCTTCCGATCTTGGTGCCATCTCCTAGAATCGAACCAGGGTTTCATCGGCCGATCGTCGGACTGTAGAACTCTGAAC-3‘ |
|  |  | Ile  Ile | AAT  TAT | 5‘-AGACGTGTGCTCTTCCGATCTTGGTGGTCTCTAGCGGGATCGAACCGCTGATCCCCGCGTTGATCGTCGGACTGTAGAACTCTGAAC-3‘  5‘-AGACGTGTGCTCTTCCGATCTTGGTGCTCGAGGTGGGGTTTGAACCCACGACGGTCGCGTTGATCGTCGGACTGTAGAACTCTGAAC-3‘ |
|  |  | Leu Leu Leu  Leu | GAG TAA CAA  TAG | 5‘-AGACGTGTGCTCTTCCGATCTTGGTGATACCCGCGGGGTTTGAACCCGCGCCTCCGAAGAGGATCGTCGGACTGTAGAACTCTGAAC-3‘  5‘-AGACGTGTGCTCTTCCGATCTTGGTGAAGGATGCGAGGTTCGAACTCGCGCGGACAACCGTGATCGTCGGACTGTAGAACTCTGAAC-3‘  5‘-AGACGTGTGCTCTTCCGATCTTGGTGGTTGCTAAGAGATTCGAACTCTTGCATCTTACGATGATCGTCGGACTGTAGAACTCTGAAC-3‘  5‘-AGACGTGTGCTCTTCCGATCTTGGTGAGAGCTAAGGGATTCGAACCCTTGCATCCGAAGATGATCGTCGGACTGTAGAACTCTGAAC-3‘ |
|  |  | Lys  Lys | TTT  CTT | 5‘-AGACGTGTGCTCTTCCGATCTTGGCTCCTCATAGGGGGCTCGAACCCCTGACATTTCGGTTGATCGTCGGACTGTAGAACTCTGAAC-3‘  5‘-AGACGTGTGCTCTTCCGATCTTGGAGCCCTGTAGGGGGCTCGAACCCCTAACCTTATGATTGATCGTCGGACTGTAGAACTCTGAAC-3‘ |
|  |  | Met | CAT | 5‘-AGACGTGTGCTCTTCCGATCTTGGTGCTCCAGGAGAGGTTCGAACTCTCGACCTTCAGATTGATCGTCGGACTGTAGAACTCTGAAC-3‘ |
|  |  | Phe | GAA | 5‘-AGACGTGTGCTCTTCCGATCTTGGTGCGAATTCTGTGGATCGAACACAGGACCTCCAGATCGATCGTCGGACTGTAGAACTCTGAAC-3‘ |
|  |  | Pro  Pro | AGG  TGG | 5‘-AGACGTGTGCTCTTCCGATCTTGGGGGGCGAGCCGGGACTCGAACCCGGGACCTCCCGCATGATCGTCGGACTGTAGAACTCTGAAC-3‘  5‘-AGACGTGTGCTCTTCCGATCTTGGGGGGCGAGCTGGGAATTGAACCCAGGGCCTCTCGCACGATCGTCGGACTGTAGAACTCTGAAC-3‘ |
|  |  | Ser Ser  Ser | GCT AGA  CGA & TGA | 5‘-AGACGTGTGCTCTTCCGATCTTGGCGTCACAGACAGGATTCGAACCTGCGCAGGTAAAACCGATCGTCGGACTGTAGAACTCTGAAC-3‘  5‘-AGACGTGTGCTCTTCCGATCTTGGCGACAACTGCAGGACTCGAACCTGCGCGGGCAAAGCCGATCGTCGGACTGTAGAACTCTGAAC-3‘  5‘-AGACGTGTGCTCTTCCGATCTTGGCGACACCAGCAGGATTTGAACCAGCGCGGGCAGAGCCGATCGTCGGACTGTAGAACTCTGAAC-3‘ |
|  |  | Thr Thr  Thr | CGT AGT  TGT | 5‘-AGACGTGTGCTCTTCCGATCTTGGTGCCCTCTGTGGGAATTGAACCCACGATCCCCGCATTGATCGTCGGACTGTAGAACTCTGAAC-3‘  5‘-AGACGTGTGCTCTTCCGATCTTGGTGCTTCCAATCGGATTTGAACCGATGATCTCCACATTGATCGTCGGACTGTAGAACTCTGAAC-3‘  5‘-AGACGTGTGCTCTTCCGATCTTGGTGCCACCTGTCAGAATTGAACTAACGACCTTTGCATTGATCGTCGGACTGTAGAACTCTGAAC-3‘ |
|  |  | Trp | CCA | 5‘-AGACGTGTGCTCTTCCGATCTTGGTGAAACGGACAGGAATTGAACCTGCAACCCTTCGATTGATCGTCGGACTGTAGAACTCTGAAC-3‘ |
|  |  | Tyr | GTA | 5‘-AGACGTGTGCTCTTCCGATCTTGGTCTCCCGGGGGCGAGTCGAACGCCCGATCTCAAGATTGATCGTCGGACTGTAGAACTCTGAAC-3‘ |
|  |  | Val  Val | CAC & TAC  AAC | 5‘-AGACGTGTGCTCTTCCGATCTTGGTGTTCCAACCGAGGATCGAACTCGGGACCTTTGCCGTGATCGTCGGACTGTAGAACTCTGAAC-3‘  5‘-AGACGTGTGCTCTTCCGATCTTGGTGATTTCGCCCAGGATCGAACTGGGGACGTTCTGCGTGATCGTCGGACTGTAGAACTCTGAAC-3‘ |

*Of note, the cDOQ His ^GTG^, contains 3 mismatches within its 40 nt sequence as a result of database ambiguity. As it still complies to our dogma (≤ 6nt levenshtein distance) for cDOQ design and can target all His-tRNA isodecoders in *S.cerevisiae*, we consider potential influences as negligable.

**Supplementary Table 4: cDOQs designed for *H. sapiens***

| *Homo sapiens* | **Type** | **tRNA** | **Anticodon** | **Sequence** |
| --- | --- | --- | --- | --- |
|  | mitochondrial | Ala | TGC | 5‘-AGACGTGTGCTCTTCCGATCTTGGTAAGGACTGCAAAACCCCACTCTGCATCAACTGAACGGATCGTCGGACTGTAGAACTCTGAAC-3‘ |
|  |  | Arg | TCG | 5‘-AGACGTGTGCTCTTCCGATCTTGGTTGGTAAATATGATTATCATAATTTAATGAGTCGAAAGATCGTCGGACTGTAGAACTCTGAAC-3‘ |
|  |  | Asn | GTT | 5‘-AGACGTGTGCTCTTCCGATCTTGGCTAGACCAATGGGACTTAAACCCACAAACACTTAGTTGATCGTCGGACTGTAGAACTCTGAAC-3‘ |
|  |  | Asp | GTC | 5‘-AGACGTGTGCTCTTCCGATCTTGGTAAGATATATAGGATTTAGCCTATAATTTAACTTTGAGATCGTCGGACTGTAGAACTCTGAAC-3‘ |
|  |  | Cys | GCA | 5‘-AGACGTGTGCTCTTCCGATCTTGGAAGCCCCGGCAGGTTTGAAGCTGCTTCTTCGAATTTGGATCGTCGGACTGTAGAACTCTGAAC-3‘ |
|  |  | Gln | TTG | 5‘-AGACGTGTGCTCTTCCGATCTTGGCTAGGACTATGAGAATCGAACCCATCCCTGAGAATCCGATCGTCGGACTGTAGAACTCTGAAC-3‘ |
|  |  | Glu | TTC | 5‘-AGACGTGTGCTCTTCCGATCTTGGTATTCTCGCACGGACTACAACCACGACCAATGATATGGATCGTCGGACTGTAGAACTCTGAAC-3‘ |
|  |  | Gly | TCC | 5‘-AGACGTGTGCTCTTCCGATCTTGGTACTCTTTTTTGAATGTTGTCAAAACTAGTTAATTGGGATCGTCGGACTGTAGAACTCTGAAC-3‘ |
|  |  | His | GTG | 5‘-AGACGTGTGCTCTTCCGATCTTGGGGTAAATAAGGGGTCGTAAGCCTCTGTTGTCAGATTCGATCGTCGGACTGTAGAACTCTGAAC-3‘ |
|  |  | Ile | GAT | 5‘-AGACGTGTGCTCTTCCGATCTTGGTAGAAATAAGGGGGTTTAAGCTCCTATTATTTACTCTGATCGTCGGACTGTAGAACTCTGAAC-3‘ |
|  |  | Leu  Leu | TAA  TAG | 5‘-AGACGTGTGCTCTTCCGATCTTGGTGTTAAGAAGAGGAATTGAACCTCTGACTGTAAAGTTGATCGTCGGACTGTAGAACTCTGAAC-3‘  5‘-AGACGTGTGCTCTTCCGATCTTGGTACTTTTATTTGGAGTTGCACCAAAATTTTTGGGGCCGATCGTCGGACTGTAGAACTCTGAAC-3‘ |
|  |  | Lys | TTT | 5‘-AGACGTGTGCTCTTCCGATCTTGGTCACTGTAAAGAGGTGTTGGTTCTCTTAATCTTTAACGATCGTCGGACTGTAGAACTCTGAAC-3‘ |
|  |  | Met | CAT | 5‘-AGACGTGTGCTCTTCCGATCTTGGTAGTACGGGAAGGGTATAACCAACATTTTCGGGGTATGATCGTCGGACTGTAGAACTCTGAAC-3‘ |
|  |  | Phe | GAA | 5‘-AGACGTGTGCTCTTCCGATCTTGGTGTTTATGGGGTGATGTGAGCCCGTCTAAACATTTTCGATCGTCGGACTGTAGAACTCTGAAC-3‘ |
|  |  | Pro | TGG | 5‘-AGACGTGTGCTCTTCCGATCTTGGTCAGAGAAAAAGTCTTTAACTCCACCATTAGCACCCAGATCGTCGGACTGTAGAACTCTGAAC-3‘ |
|  |  | Ser  Ser | GCT  TGA | 5‘-AGACGTGTGCTCTTCCGATCTTGGTGAGAAAGCCATGTTGTTAGACATGGGGGCATGAGTTGATCGTCGGACTGTAGAACTCTGAAC-3‘  5‘-AGACGTGTGCTCTTCCGATCTTGGCAAAAAAGGAAGGAATCGAACCCCCCAAAGCTGGTTTGATCGTCGGACTGTAGAACTCTGAAC-3‘ |
|  |  | Thr | TGT | 5‘-AGACGTGTGCTCTTCCGATCTTGGTGTCCTTGGAAAAAGGTTTTCATCTCCGGTTTACAAGGATCGTCGGACTGTAGAACTCTGAAC-3‘ |
|  |  | Trp | TCA | 5‘-AGACGTGTGCTCTTCCGATCTTGGCAGAAATTAAGTATTGCAACTTACTGAGGGCTTTGAAGATCGTCGGACTGTAGAACTCTGAAC-3‘ |
|  |  | Tyr | GTA | 5‘-AGACGTGTGCTCTTCCGATCTTGGGGTAAAAAGAGGCCTAACCCCTGTCTTTAGATTTACAGATCGTCGGACTGTAGAACTCTGAAC-3‘ |
|  |  | Val | TAC | 5‘-AGACGTGTGCTCTTCCGATCTTGGTCAGAGCGGTCAAGTTAAGTTGAAATCTCCTAAGTGTGATCGTCGGACTGTAGAACTCTGAAC-3‘ |
|  | cytosolic | Ala  Ala | AGC  CGC & TGC | 5‘-AGACGTGTGCTCTTCCGATCTTGGTGGAGAATGCGGGCATCGATCCCGCTACCTCTCGCATGATCGTCGGACTGTAGAACTCTGAAC-3‘  5‘-AGACGTGTGCTCTTCCGATCTTGGTGGAGGTGCCGGGGATCGAACCCGGGGCCTCATACATGATCGTCGGACTGTAGAACTCTGAAC-3‘ |
|  |  | Arg Arg Arg  Arg | ACG CCG CCT  TCT | 5‘-AGACGTGTGCTCTTCCGATCTTGGCGAGCCAGCCAGGAGTCGAACCTAGAATCTTCTGATCGATCGTCGGACTGTAGAACTCTGAAC-3‘  5‘-AGACGTGTGCTCTTCCGATCTTGGCGACCACGAAGGGACTCGAACCCTCAATCTTCTGATCGATCGTCGGACTGTAGAACTCTGAAC-3‘  5‘-AGACGTGTGCTCTTCCGATCTTGGCACCCCAGGTGGGACTCGAACCCACAATCCCTGGCTTGATCGTCGGACTGTAGAACTCTGAAC-3‘  5‘-AGACGTGTGCTCTTCCGATCTTGGCGACTCTGCCGGGACTCGAACCCGCAACCTTTGAATTGATCGTCGGACTGTAGAACTCTGAAC-3‘ |
|  |  | Asn | GTT | 5‘-AGACGTGTGCTCTTCCGATCTTGGCGTCCCTGGGTGGGCTCGAACCACCAACCTTTCGGTTGATCGTCGGACTGTAGAACTCTGAAC-3‘ |
|  |  | Asp | GTC | 5‘-AGACGTGTGCTCTTCCGATCTTGGCTCCCCGTCGGGGAATCGAACCCCGGTCTCCCGCGTGGATCGTCGGACTGTAGAACTCTGAAC-3‘ |
|  |  | Cys | GCA | 5‘-AGACGTGTGCTCTTCCGATCTTGGAGGGGGCACCCGGATTTGAACCAGGGACCTCTTGATCGATCGTCGGACTGTAGAACTCTGAAC-3‘ |
|  |  | iMet | CAT | 5‘-AGACGTGTGCTCTTCCGATCTTGGTAGCAGAGGATGGTTTCGATCCATCGACCTCTGGGTTGATCGTCGGACTGTAGAACTCTGAAC-3‘ |
|  |  | Gln | TTG & CTG | 5‘-AGACGTGTGCTCTTCCGATCTTGGAGGTTCCACCGAGATTTGAACTCGGATCGCTGGATTCGATCGTCGGACTGTAGAACTCTGAAC-3‘ |
|  |  | Glu  Glu | CTC & TTC (1)  TTC (2) | 5‘-AGACGTGTGCTCTTCCGATCTTGGTTCCCTGACCGGGAATCGAACCCGGGCCGCGGCGGTGGATCGTCGGACTGTAGAACTCTGAAC-3‘  5‘-AGACGTGTGCTCTTCCGATCTTGGTTCCCACACCGGGAGTCGAACCCGGGCCGCCTGGGTGGATCGTCGGACTGTAGAACTCTGAAC-3‘ |
|  |  | Gly Gly  Gly | CCC (1) GCC & CCC (2)  TCC | 5‘-AGACGTGTGCTCTTCCGATCTTGGTGCGCCGCCCGGGAATCGAACCCGGGTCGCAAGAATGGATCGTCGGACTGTAGAACTCTGAAC-3‘  5‘-AGACGTGTGCTCTTCCGATCTTGGTGCATTGGCCGGGAATCGAACCCGGGCCTCCCGCGTGGATCGTCGGACTGTAGAACTCTGAAC-3‘  5‘-AGACGTGTGCTCTTCCGATCTTGGTGCGTTGGCCGGGAATCGAACCCGGGTCAACTGCTTGGATCGTCGGACTGTAGAACTCTGAAC-3‘ |
|  |  | His | GTG | 5‘-AGACGTGTGCTCTTCCGATCTTGGTGCCGTGACTCGGATTCGAACCGAGGTTGCTGCGGCCGATCGTCGGACTGTAGAACTCTGAAC-3‘ |

| *Homo sapiens* | **Type** | **tRNA** | **Anticodon** | **Sequence** |
| --- | --- | --- | --- | --- |
|  | cytosolic | Ile | GAT & AAT | 5‘-AGACGTGTGCTCTTCCGATCTTGGTGGCCCGTACGGGGATCGAACCCGCGACCTTGGCGTTGATCGTCGGACTGTAGAACTCTGAAC-3‘ |
|  |  | Ile | TAT | 5‘-AGACGTGTGCTCTTCCGATCTTGGTGCTCCAGGTGAGGCTCGAACTCACAACCTCGGCATTGATCGTCGGACTGTAGAACTCTGAAC-3‘ |
|  |  | Leu | TAG & AAG | 5‘-AGACGTGTGCTCTTCCGATCTTGGTGGCAGCGGTGGGATTCGAACCCACGCCCCCGAAGAGGATCGTCGGACTGTAGAACTCTGAAC-3‘ |
|  |  | Leu Leu  Leu | CAA (1) CAG & CAA (2)  TAA | 5‘-AGACGTGTGCTCTTCCGATCTTGGTGCCCCCTCTGAGGCTTGAACTCAGGACCATTCAGATGATCGTCGGACTGTAGAACTCTGAAC-3‘  5‘-AGACGTGTGCTCTTCCGATCTTGGTGTCAGAAGTGGGATTCGAACCCACGCCTCCATCGGAGATCGTCGGACTGTAGAACTCTGAAC-3‘  5‘-AGACGTGTGCTCTTCCGATCTTGGTACCAGGAGTGGGGTTCGAACCCACGCGGACACCAGTGATCGTCGGACTGTAGAACTCTGAAC-3‘ |
|  |  | Lys | CTT | 5‘-AGACGTGTGCTCTTCCGATCTTGGCGCCCAACGTGGGGCTCGAACCCACGACCCTGAGATTGATCGTCGGACTGTAGAACTCTGAAC-3‘ |
|  |  | Lys | TTT | 5‘-AGACGTGTGCTCTTCCGATCTTGGCGCCTGAACAGGGACTTGAACCCTGGACCCTCAGATTGATCGTCGGACTGTAGAACTCTGAAC-3‘ |
|  |  | Met | CAT | 5‘-AGACGTGTGCTCTTCCGATCTTGGTGCCCTCTCTGAGGCTCGAACTCAGGACCTTCAGATTGATCGTCGGACTGTAGAACTCTGAAC-3‘ |
|  |  | Phe | GAA | 5‘-AGACGTGTGCTCTTCCGATCTTGGTGCCGAAACCCGGGATTGAACCAGGGACCTTTAGATCGATCGTCGGACTGTAGAACTCTGAAC-3‘ |
|  |  | Pro | TGG & CGG & AGG | 5‘-AGACGTGTGCTCTTCCGATCTTGGGGGCTCGTCCGGGATTTGAACCCGGGACCTCTCGCACGATCGTCGGACTGTAGAACTCTGAAC-3‘ |
|  |  | Sec | TCA | 5‘-AGACGTGTGCTCTTCCGATCTTGGCGCCCGAAAGGTGGAATTGAACCACTCTGTCGCTAGAGATCGTCGGACTGTAGAACTCTGAAC-3‘ |
|  |  | Ser | TGA & AGA | 5‘-AGACGTGTGCTCTTCCGATCTTGGCGTAGTCGGCAGGATTCGAACCTGCGCGGGGAGACCCGATCGTCGGACTGTAGAACTCTGAAC-3‘ |
|  |  | Ser Ser  Ser | CGA (1)  CGA (2)  GCT | 5‘-AGACGTGTGCTCTTCCGATCTTGGCGCTGTGAGCAGGATTTGAACCTGCGCGGGGAGACCCGATCGTCGGACTGTAGAACTCTGAAC-3‘  5‘-AGACGTGTGCTCTTCCGATCTTGGCGTCACGAACAGGATTCGAACCTGTGCGGGGAAACCCGATCGTCGGACTGTAGAACTCTGAAC-3‘  5‘-AGACGTGTGCTCTTCCGATCTTGGCGACGAGGATGGGATTCGAACCCACGCGTGCAGAGCAGATCGTCGGACTGTAGAACTCTGAAC-3‘ |
|  |  | Thr | AGT & TGT (1) & CGT (1) | 5‘-AGACGTGTGCTCTTCCGATCTTGGAGGCCCCGCTGGGATTCGAACCCAGGATCTCCTGTTTGATCGTCGGACTGTAGAACTCTGAAC-3‘ |
|  |  | Thr Thr | TGT (2) & CGT (2)  CGT (3) | 5‘-AGACGTGTGCTCTTCCGATCTTGGAGGCCCCAGCGAGATTTGAACTCGCGACCCCTGGTTTGATCGTCGGACTGTAGAACTCTGAAC-3‘  5‘-AGACGTGTGCTCTTCCGATCTTGGAGGCACGGACGGGGTTCGAACCCGTGATCTTCGGTTTGATCGTCGGACTGTAGAACTCTGAAC-3‘ |
|  |  | Trp | CCA | 5‘-AGACGTGTGCTCTTCCGATCTTGGTGACCCCGACGTGATTTGAACACGCAACCTTCTGATCGATCGTCGGACTGTAGAACTCTGAAC-3‘ |
|  |  | Tyr | ATA | 5‘-AGACGTGTGCTCTTCCGATCTTGGTCCTTCAAGCTGGAATCGAACCAGCAACCTAAGGACCGATCGTCGGACTGTAGAACTCTGAAC-3‘ |
|  |  | Tyr Tyr | GTA (1)  GTA (2) | 5‘-AGACGTGTGCTCTTCCGATCTTGGTCCTTCGAGCCGGAATCGAACCAGCGACCTAAGGATCGATCGTCGGACTGTAGAACTCTGAAC-3‘  5‘-AGACGTGTGCTCTTCCGATCTTGGCTCTCCAAGTCGGAATCAAACCAGCACCTAAGAATCTGATCGTCGGACTGTAGAACTCTGAAC-3‘ |
|  |  | Val | CAC & TAC (1) & AAC (1) | 5‘-AGACGTGTGCTCTTCCGATCTTGGTGTTTCCGCCCGGTTTCGAACCGGGGACCTTTCGCGTGATCGTCGGACTGTAGAACTCTGAAC-3‘ |
|  |  | Val  Val | AAC (2)  TAC (2) | 5‘-AGACGTGTGCTCTTCCGATCTTGGTGGAAGTGCTGGGGATCGAACCCAGAGCCTCATGAATGATCGTCGGACTGTAGAACTCTGAAC-3‘  5‘-AGACGTGTGCTCTTCCGATCTTGGTGGTTCCACTGGGGCTCGAACCCAGGACCTTCTGCGTGATCGTCGGACTGTAGAACTCTGAAC-3‘ |

**Supplementary Table 5: cDOQs designed for *M. musculus***

| *Mus musculus* | **Type** | **tRNA** | **Anticodon (* = Same cDOQ** | **Sequence** |
| --- | --- | --- | --- | --- |
|  |  |  | **as *H. sapiens*)** |  |
|  | mitochondrial | Ala | TGC | 5‘-AGACGTGTGCTCTTCCGATCTTGGTCAGAGTGTTCATTGGTCATGAAATCTTCTGGGTGTAGATCGTCGGACTGTAGAACTCTGAAC-3‘ |
|  |  | Arg | TCG | 5‘-AGACGTGTGCTCTTCCGATCTTGGTGGTAAAAAGAGGATTTAAACCTCTGTGTTTAGATTTGATCGTCGGACTGTAGAACTCTGAAC-3‘ |
|  |  | Asn | GTT | 5‘-AGACGTGTGCTCTTCCGATCTTGGCAGAAGTTAAACTTGTGTGTTTTCTTAGGGCTTTGAAGATCGTCGGACTGTAGAACTCTGAAC-3‘ |
|  |  | Asp | GTC | 5‘-AGACGTGTGCTCTTCCGATCTTGGTGTCTTGAGAAGAGAAGATCTTCATTTCAGGTTTACAGATCGTCGGACTGTAGAACTCTGAAC-3‘ |
|  |  | Cys | GCA | 5‘-AGACGTGTGCTCTTCCGATCTTGGTAAGAAAGGAAGGAATCGAACCCCCTAAAATTGGTTTGATCGTCGGACTGTAGAACTCTGAAC-3‘ |
|  |  | Gln | TTG | 5‘-AGACGTGTGCTCTTCCGATCTTGGTAAGAAAGCCATGTTTTTAAACATGGAAGCATGAATTGATCGTCGGACTGTAGAACTCTGAAC-3‘ |
|  |  | Glu | TTC | 5‘-AGACGTGTGCTCTTCCGATCTTGGTCAAGAAGAAGGAGCTACTCCCCACCACCAGCACCCAGATCGTCGGACTGTAGAACTCTGAAC-3‘ |
|  |  | Gly | TCC | 5‘-AGACGTGTGCTCTTCCGATCTTGGTGTTTATGGGATACAATTATCCATCTAAGCATTTTCAGATCGTCGGACTGTAGAACTCTGAAC-3‘ |
|  |  | His | GTG | 5‘-AGACGTGTGCTCTTCCGATCTTGGTAGTACGGGAAGGATTTAAACCAACGTTTTCGGGGTAGATCGTCGGACTGTAGAACTCTGAAC-3‘ |
|  |  | Ile | GAT | 5‘-AGACGTGTGCTCTTCCGATCTTGGTCACTATGGAGATTTTAAGGTCTCTAACTTTAACTTAGATCGTCGGACTGTAGAACTCTGAAC-3‘ |
|  |  | Leu | TAA | 5‘-AGACGTGTGCTCTTCCGATCTTGGTATTAGGGAGAGGATTTGAACCTCTGGGAACAAGGTTGATCGTCGGACTGTAGAACTCTGAAC-3‘ |
|  |  | Leu | TAG | 5‘-AGACGTGTGCTCTTCCGATCTTGGTACTTTTATTTGGATTTGCACCAAGGTTTTTGGTTCCGATCGTCGGACTGTAGAACTCTGAAC-3‘ |
|  |  | Lys | TTT | 5‘-AGACGTGTGCTCTTCCGATCTTGGTAGAAATAAGAGGGCTTGAACCTCTATAATTTACTCTGATCGTCGGACTGTAGAACTCTGAAC-3‘ |
|  |  | Met | CAT | 5‘-AGACGTGTGCTCTTCCGATCTTGGGGTGAATAAGGAGGTTTATTTCCTGTTGTCAGATTCAGATCGTCGGACTGTAGAACTCTGAAC-3‘ |
|  |  | Phe | GAA | 5‘-AGACGTGTGCTCTTCCGATCTTGGTACTCTCTTCTGGGTTTATTCAGAATCTACTAATTGGGATCGTCGGACTGTAGAACTCTGAAC-3‘ |
|  |  | Pro | TGG | 5‘-AGACGTGTGCTCTTCCGATCTTGGTATTTCTACACAGCATTCAACTGCGACCAATGACATGGATCGTCGGACTGTAGAACTCTGAAC-3‘ |
|  |  | Ser | GCT | 5‘-AGACGTGTGCTCTTCCGATCTTGGCTAGGACAATAGGAATTGAACCTACACTTAAGAATTCGATCGTCGGACTGTAGAACTCTGAAC-3‘ |
|  |  | Ser | TGA | 5‘-AGACGTGTGCTCTTCCGATCTTGGAAGTCTTAGTAGAGATTTCTCTACACCTTCGAATTTGGATCGTCGGACTGTAGAACTCTGAAC-3‘ |
|  |  | Thr | TGT | 5‘-AGACGTGTGCTCTTCCGATCTTGGTAAGATATATAGATTATTGATCTATAATTTAACTTTGGATCGTCGGACTGTAGAACTCTGAAC-3‘ |
|  |  | Trp | TCA | 5‘-AGACGTGTGCTCTTCCGATCTTGGCTAGATTGGCAGGAATTAAACCTACGAAAATTTAGTTGATCGTCGGACTGTAGAACTCTGAAC-3‘ |
|  |  | Tyr | GTA | 5‘-AGACGTGTGCTCTTCCGATCTTGGTTGGTAATTATGAACATCATCATAATCTAATGAGTCGGATCGTCGGACTGTAGAACTCTGAAC-3‘ |
|  |  | Val | TAC | 5‘-AGACGTGTGCTCTTCCGATCTTGGTAAGGACTGTAAGACTTCATCCTACATCTATTGAATGGATCGTCGGACTGTAGAACTCTGAAC-3‘ |
|  | cytosolic | Ala | AGC | 5‘-AGACGTGTGCTCTTCCGATCTTGGAAAAAAACCTGGAGATCGAACCCAGGACCTCATGCATGATCGTCGGACTGTAGAACTCTGAAC-3‘ |
|  |  | Ala | TGC & CGC (1)* | 5‘-AGACGTGTGCTCTTCCGATCTTGGTGGAGGTGCCGGGGATCGAACCCGGGGCCTCATACATGATCGTCGGACTGTAGAACTCTGAAC-3‘ |
|  |  | Ala | CGC (2) | 5‘-AGACGTGTGCTCTTCCGATCTTGGTTCCCTGACTAGGATTCAAACCTGGGCCACGGCGGTGGATCGTCGGACTGTAGAACTCTGAAC-3‘ |
|  |  | Arg | ACG | 5‘-AGACGTGTGCTCTTCCGATCTTGGCGAGCCAGCCAGGAGTCGAACCTAGAATCTTCTGATCGATCGTCGGACTGTAGAACTCTGAAC-3‘ |
|  |  | Arg | CCT | 5‘-AGACGTGTGCTCTTCCGATCTTGGTACCCCAGGTGGGACTCGAACCCACAATCCCTGGCTTGATCGTCGGACTGTAGAACTCTGAAC-3‘ |
|  |  | Arg | TCG & CCG | 5‘-AGACGTGTGCTCTTCCGATCTTGGCGACCGCGCCAGGACTCGAACCTGCAATCTTCTGATCGATCGTCGGACTGTAGAACTCTGAAC-3‘ |
|  |  | Arg | TCT (1) | 5‘-AGACGTGTGCTCTTCCGATCTTGGCATCTCTGCCGGGACTCGAACCCAGAACCTCTGGATTGATCGTCGGACTGTAGAACTCTGAAC-3‘ |
|  |  | Arg | TCT (2) | 5‘-AGACGTGTGCTCTTCCGATCTTGGCGACTCTGCCGGGACTCGAACCCGCAACCTTTGAATTGATCGTCGGACTGTAGAACTCTGAAC-3‘ |
|  |  | Asn | GTT* | 5‘-AGACGTGTGCTCTTCCGATCTTGGCGTCCCTGGGTGGGCTCGAACCACCAACCTTTCGGTTGATCGTCGGACTGTAGAACTCTGAAC-3‘ |
|  |  | Asp | GTC* | 5‘-AGACGTGTGCTCTTCCGATCTTGGCTCCCCGTCGGGGAATCGAACCCCGGTCTCCCGCGTGGATCGTCGGACTGTAGAACTCTGAAC-3‘ |
|  |  | Cys | GCA* | 5‘-AGACGTGTGCTCTTCCGATCTTGGAGGGGGCACCCGGATTTGAACCAGGGACCTCTTGATCGATCGTCGGACTGTAGAACTCTGAAC-3‘ |
|  |  | iMet | CAT* | 5‘-AGACGTGTGCTCTTCCGATCTTGGTAGCAGAGGATGGTTTCGATCCATCGACCTCTGGGTTGATCGTCGGACTGTAGAACTCTGAAC-3‘ |
|  |  | Gln | TTG & CTG* | 5‘-AGACGTGTGCTCTTCCGATCTTGGAGGTTCCACCGAGATTTGAACTCGGATCGCTGGATTCGATCGTCGGACTGTAGAACTCTGAAC-3‘ |
|  |  | Glu | TTC & CTC (1)* | 5‘-AGACGTGTGCTCTTCCGATCTTGGTTCCCTGACCGGGAATCGAACCCGGGCCGCGGCGGTGGATCGTCGGACTGTAGAACTCTGAAC-3‘ |
|  |  | Glu | CTC (2) | 5‘-AGACGTGTGCTCTTCCGATCTTGGTTCCCTGACCAGGAATCAAACCTGGGCCATGGAGGTGGATCGTCGGACTGTAGAACTCTGAAC-3‘ |

| *Mus musculus* | **Type** | **tRNA** | **Anticodon (* = Same**  **cDOQ as *H. sapiens*)** | **Sequence** |
| --- | --- | --- | --- | --- |
|  | cytosolic | Gly Gly  Gly | CCC (1)* TCC & CCC (2)*  GCC | 5‘-AGACGTGTGCTCTTCCGATCTTGGTGCGCCGCCCGGGAATCGAACCCGGGTCGCAAGAATGGATCGTCGGACTGTAGAACTCTGAAC-3‘  5‘-AGACGTGTGCTCTTCCGATCTTGGTGCATTGGCCGGGAATCGAACCCGGGCCTCCCGCGTGGATCGTCGGACTGTAGAACTCTGAAC-3‘  5‘-AGACGTGTGCTCTTCCGATCTTGGTGTTTCCGCCCGGTTTCGAACCGGGGACCTTTCGCTTGATCGTCGGACTGTAGAACTCTGAAC-3‘ |
|  |  | His | GTG* | 5‘-AGACGTGTGCTCTTCCGATCTTGGTGCCGTGACTCGGATTCGAACCGAGGTTGCTGCGGCCGATCGTCGGACTGTAGAACTCTGAAC-3‘ |
|  |  | Ile  Ile | AAT*  TAT | 5‘-AGACGTGTGCTCTTCCGATCTTGGTGGCCCGTACGGGGATCGAACCCGCGACCTTGGCGTTGATCGTCGGACTGTAGAACTCTGAAC-3‘  5‘-AGACGTGTGCTCTTCCGATCTTGGTAGTTCCACTGGGGCTCAAACTCAGGACCTTCTGCATGATCGTCGGACTGTAGAACTCTGAAC-3‘ |
|  |  | Leu | TAG & AAG* | 5‘-AGACGTGTGCTCTTCCGATCTTGGTGGCAGCGGTGGGATTCGAACCCACGCCCCCGAAGAGGATCGTCGGACTGTAGAACTCTGAAC-3‘ |
|  |  | Leu | CAG & CAA* | 5‘-AGACGTGTGCTCTTCCGATCTTGGTGTCAGAAGTGGGATTCGAACCCACGCCTCCATCGGAGATCGTCGGACTGTAGAACTCTGAAC-3‘ |
|  |  | Leu | TAA | 5‘-AGACGTGTGCTCTTCCGATCTTGGTACTGGGAGTGGGGTTCGAACCCACGCAGGCACCAGCGATCGTCGGACTGTAGAACTCTGAAC-3‘ |
|  |  | Lys | CTT | 5‘-AGACGTGTGCTCTTCCGATCTTGGTGCCTAATATGGGGCTCGAACCCATGACCCTGGGATTGATCGTCGGACTGTAGAACTCTGAAC-3‘ |
|  |  | Lys | TTT* | 5‘-AGACGTGTGCTCTTCCGATCTTGGCGCCTGAACAGGGACTTGAACCCTGGACCCTCAGATTGATCGTCGGACTGTAGAACTCTGAAC-3‘ |
|  |  | Met | CAT | 5‘-AGACGTGTGCTCTTCCGATCTTGGTGCCCCGTGTGAGGATCGAACTCACGACCTTCAGATTGATCGTCGGACTGTAGAACTCTGAAC-3‘ |
|  |  | Phe | GAA* | 5‘-AGACGTGTGCTCTTCCGATCTTGGTGCCGAAACCCGGGATTGAACCAGGGACCTTTAGATCGATCGTCGGACTGTAGAACTCTGAAC-3‘ |
|  |  | Pro | TGG & CGG & AGG* | 5‘-AGACGTGTGCTCTTCCGATCTTGGGGGCTCGTCCGGGATTTGAACCCGGGACCTCTCGCACGATCGTCGGACTGTAGAACTCTGAAC-3‘ |
|  |  | Sec | TCA* | 5‘-AGACGTGTGCTCTTCCGATCTTGGCGCCCGAAAGGTGGAATTGAACCACTCTGTCGCTAGAGATCGTCGGACTGTAGAACTCTGAAC-3‘ |
|  |  | Ser | TGA & AGA* | 5‘-AGACGTGTGCTCTTCCGATCTTGGCGTAGTCGGCAGGATTCGAACCTGCGCGGGGAGACCCGATCGTCGGACTGTAGAACTCTGAAC-3‘ |
|  |  | Ser | CGA (1)* | 5‘-AGACGTGTGCTCTTCCGATCTTGGCGCTGTGAGCAGGATTTGAACCTGCGCGGGGAGACCCGATCGTCGGACTGTAGAACTCTGAAC-3‘ |
|  |  | Ser | CGA (2)* | 5‘-AGACGTGTGCTCTTCCGATCTTGGCGTCACGAACAGGATTCGAACCTGTGCGGGGAAACCCGATCGTCGGACTGTAGAACTCTGAAC-3‘ |
|  |  | Ser | GCT | 5‘-AGACGTGTGCTCTTCCGATCTTGGCGACGAGGATGGGATTCGAACCCACGCGTGCAAAGCAGATCGTCGGACTGTAGAACTCTGAAC-3‘ |
|  |  | Thr | AGT & TGT (1) & CGT (1)* | 5‘-AGACGTGTGCTCTTCCGATCTTGGAGGCCCCGCTGGGATTCGAACCCAGGATCTCCTGTTTGATCGTCGGACTGTAGAACTCTGAAC-3‘ |
|  |  | Thr | TGT (2) & CGT (2)* | 5‘-AGACGTGTGCTCTTCCGATCTTGGAGGCCCCAGCGAGATTTGAACTCGCGACCCCTGGTTTGATCGTCGGACTGTAGAACTCTGAAC-3‘ |
|  |  | Thr | CGT* | 5‘-AGACGTGTGCTCTTCCGATCTTGGAGGCACGGACGGGGTTCGAACCCGTGATCTTCGGTTTGATCGTCGGACTGTAGAACTCTGAAC-3‘ |
|  |  | Trp | CCA* | 5‘-AGACGTGTGCTCTTCCGATCTTGGTGACCCCGACGTGATTTGAACACGCAACCTTCTGATCGATCGTCGGACTGTAGAACTCTGAAC-3‘ |
|  |  | Tyr | GTA* | 5‘-AGACGTGTGCTCTTCCGATCTTGGTCCTTCGAGCCGGAATCGAACCAGCGACCTAAGGATCGATCGTCGGACTGTAGAACTCTGAAC-3‘ |
|  |  | Val | CAC & TAC (1) & AAC (1)* | 5‘-AGACGTGTGCTCTTCCGATCTTGGTGTTTCCGCCCGGTTTCGAACCGGGGACCTTTCGCGTGATCGTCGGACTGTAGAACTCTGAAC-3‘ |
|  |  | Val | AAC (2) | 5‘-AGACGTGTGCTCTTCCGATCTTGGCTATGGTACTGGGAATTGAACCCAGGACTTTTGCATGGATCGTCGGACTGTAGAACTCTGAAC-3‘ |
|  |  | Val | TAC (2)* | 5‘-AGACGTGTGCTCTTCCGATCTTGGTGGTTCCACTGGGGCTCGAACCCAGGACCTTCTGCGTGATCGTCGGACTGTAGAACTCTGAAC-3‘ |

**Supplementary Table 6: Comparative table of exemplary tRNA quantification methods**

| **Name, if applicable** | **Author** | **Published** | **Method** | **Amount** | **Parallel quantification of all tRNAs?** | **Enzymatic steps** | **DOI** |
| --- | --- | --- | --- | --- | --- | --- | --- |
| mim-tRNAseq | Behrens *et al.* | 2021 | RNAseq | 50-200 ng total tRNA | Yes | 6 | 10.1016/j.molcel.2021.01.028 |
| OTTER | Nagai *et al.* | 2021 | In gel fluorescence | 2 µg total RNA | No | 1 | 10.1261/rna.076489.120 |
| AQRNAseq | Hu *et al.* | 2021 | RNAseq | 50 ng total tRNA | Yes | 10 | 10.1038/s41587-021-00874-y |
| QuantM-tRNA-seq | Pinkard *et al.* | 2020 | RNAseq | 1 µg deacetyl. total RNA | Yes | 5 | 10.1038/s41467-020-17879-x |
| MST | Jacob *et al.* | 2019 | Microscale Thermophoresis | 1-2 µg tot tRNA | No | 0 | 10.1002/anie.201814377 |
| tRNA microarray | Polte *et al.* | 2018 | Microarray | 1-2 µg fluoro labeled tRNA | No | 1 | 10.1186/s12864-019-5864-1 |
| Hydro-tRNAseq | Gogakos *et al.* | 2017 | RNAseq | 20 µg total RNA | Yes | 6 | 10.1016/j.celrep.2017.07.029 |
| YAMAT-seq | Shigematsu *et al.* | 2017 | RNAseq | 1 µg deac. total RNA | Yes | 3 | 10.1093/nar/gkx005 |
| DM-TGIRT-seq | Zheng *et al.* | 2015 | RNAseq + alkB | 100 ng demethyl. tRNA or 1 µg tot. RNA | Yes | 4 | 10.1038/nmeth.3478 |
| ARM-seq | Cozen *et al.* | 2015 | RNAseq + alkB | 1 µg total RNA | Yes | 7 | 10.1038/nmeth.3508 |
| tRNA-seq | Pang *et al.* | 2014 | RNAseq | - | Yes | 6 | 10.1093/nar/gku945 |
| tRNA-microarrays | Dittmar *et al.* | 2006 | Microarray | 1 µg fluoro labeled total RNA | No | 1 | 10.1371/journal.pgen.0020221 |
| - | Dong *et al.* | 1996 | 2D gel radiolabel | 5 µg total RNA | No | 0 | 10.1006/jmbi.1996.0428 |
| - | Ikemura *et al.* | 1982 | 2D gel radiolabel | - | No | 0 | 10.1016/0022-2836(82)90250-9 |
| Our method | Kristen *et al.* | - | Hybridization + NGS | 5-25 ng total tRNA | Yes | 1 | - |
